# Zinc systematics quantify crustal thickness control on fractionating assemblages of arc magmas

**DOI:** 10.1038/s41598-021-94290-6

**Published:** 2021-07-19

**Authors:** M. Chiaradia

**Affiliations:** Department of Earth Sciences, University of Geneva, Rue des Maraîchers 13, 1205 Geneva, Switzerland

**Keywords:** Planetary science, Geochemistry

## Abstract

Understanding the processes leading to the broad chemical variability of arc magmas is an essential, yet not fully elucidated, issue in Earth Sciences. Here, I show that Zn–MgO–SiO_2_ systematics of magmatic arc rocks correlate significantly with arc thickness. Because Zn–MgO–SiO_2_ systematics are mostly controlled by fractionation of different mineral phases, this suggests a systematic change in the proportions of fractionating mineral assemblages depending on arc thickness. Using a mass balance model with a Monte Carlo approach, I show that Zn–MgO–SiO_2_ systematics can be quantitatively explained by a continuous transition from plagioclase-dominated fractionating assemblages in thin arcs to amphibole-garnet-magnetite-dominated assemblages in increasingly thicker arcs. Most likely, such a systematic change results from the increase of average depth of magma differentiation that is ultimately controlled by arc thickness. Results presented have implications on the causes of different geochemical trends in arcs, the role of arcs as H_2_O filters, and their association with porphyry deposits.

## Introduction

Arc magmas are the building blocks of the continental crust^1^ and are associated with the formation of economic mineral deposits^2–4^ as well as with catastrophic eruptions impacting human lives^5^. They are also key to understand the recycling of elements and volatiles from the Earth crust and surface to the deep mantle because they represent the output of such interaction in the mantle wedge^6^. In recent years various studies have shown systematic correlations of major and trace elements of arc magmas with the crustal thickness of the overriding plate^7–11^. However, it is not clear to what extent such correlations are controlled by mantle wedge or by intracrustal processes. For instance, it has been shown that the development of distinct tholeiitic and calc-alkaline trends in arc magmas depends on crustal thickness^12^, but this has been interpreted either as the result of crustal processes^7,13^ or of different degrees of partial melting of the mantle wedge^14^. The latter would be due to a different thermal structure of the mantle wedge that is controlled by the thickness of the overriding plate crust and lithosphere^15^. Least evolved rocks of arcs have also been shown to display systematic correlations of their major and trace element contents with crustal thickness^10,11^. This too has been interpreted as the result of differential partial melting degrees of the mantle wedge depending on the different depths of mantle wedge melting^10,11^, which are ultimately controlled by the thickness of the overriding plate crust.

In this study I use Zn systematics to investigate the role of fractional crystallization in producing different geochemical signatures and evolutionary trends in arc magmas and their relationship with the crustal thickness of the overriding plate. Zn is a lithophile element that has received an increased attention in magmatic processes during the last years because it is used as a reference element (e.g., Zn/Fe ratios) for tracing the oxidation state of the mantle^16^, it allows investigation of mantle melting processes^17^, and because Zn isotopes may elucidate processes of magma differentiation^18^, slab contributions^19^, and planetary evolution^20^. Zinc is incorporated to different extents into the crystal lattice of all main minerals crystallizing from arc magmas (olivine, pyroxene, amphibole, plagioclase, magnetite, and garnet)^17^. This property, together with systematics of major elements like SiO_2_ and MgO, which are also sensitive to fractionation of major mineral phases, may be used to reveal and quantify changes in the assemblages fractionating during arc magma evolution. It can also improve our understanding of the processes through which crustal thickness determines the development of different geochemical signatures and trends in arc magmas. The advantage of using Zn over other elements, typically used to evaluate fractionation of mineral assemblages in arc magmas (e.g., REE, Sr, Y, Cu), is that Zn is not incorporated to high extent into accessory minerals (zircon, titanite, apatite, garnet, not even sulfide minerals: refs.^21,22^) that may strongly bias the systematics of these elements.

Here, I show that arc magmas display Zn–MgO and MgO–SiO_2_ trends that systematically change with crustal thickness. Using a Monte Carlo-based modelling of fractionating assemblages in the SiO_2_–MgO–Zn tridimensional space I show that the systematic changes in the Zn–MgO and MgO–SiO_2_ trends are controlled by fractionating assemblages that shift from plagioclase-dominated in thin arcs to amphibole-garnet-magnetite-dominated in thick arcs, and quantify the proportions of fractionating minerals. The amphibole plus garnet proportions on one hand and plagioclase proportions on the other also correlate with the tholeiitic versus calc-alkaline features of arc magmas showing that there is a gradual and continuous transition between two geochemical and mineralogical extremes, which is ultimately controlled by the different depths at which average magma differentiation occurs in arcs of different thickness.

## Results

### Data collection and treatment

Geochemical data of bulk volcanic rocks from 21 modern arcs (Supplementary Data File 1) were collected from the Georoc database (http://georoc.mpch-mainz.gwdg.de/georoc/) and treated according to the method described by ref.^7^ and detailed in the Methods section. As in ref.^7^, to reduce the bias induced by outliers and to extract information on general trends, median values of Zn, SiO_2_, and MgO for subpopulations corresponding to bins of 0.5 wt% MgO were calculated (Supplementary Data File 1). When less than 10 data were available for one of the investigated elements within the 0.5 wt% MgO bin, the MgO interval was extended to a higher value (e.g., 1 or 1.5 wt%) to incorporate more values of that element. For comparison with arc systematics, data were also collected for the mid-ocean ridge (MOR) environment (https://www.earthchem.org/) and for a typical oceanic island basalt magmatic sequence like Hawaii (http://georoc.mpch-mainz.gwdg.de/georoc/), and treated in the same way as for arcs.

**Figure 1:**
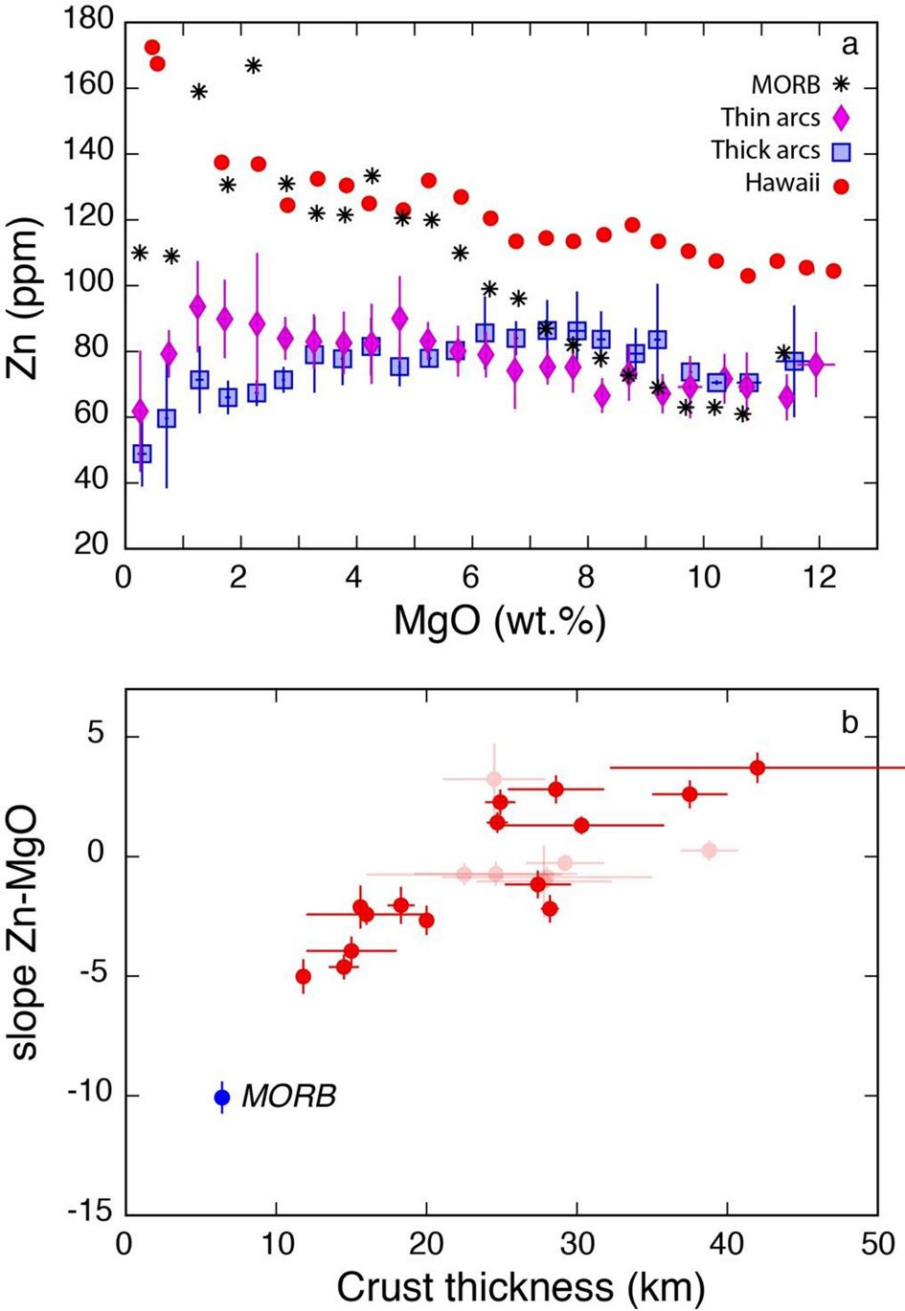
Zn–MgO systematics of arc, MOR and Hawaii magmatic rocks. (**a**) Zn–MgO plot of the averages of median values of all thin (< 20 km) and all thick (> 30 km) arcs compared to median values of MOR and Hawaii magmas for MgO bins ~ 0.5 wt% (data reported in Supplementary Data File 1); (**b**) plot of the Zn–MgO slopes of individual arcs and MOR magmatic rocks versus crustal thickness (data reported in Table 1). The light red symbols denote poor statistical significance for the Zn–MgO regressions (see Table 1).

Median values of zinc contents of magmas from different arcs follow distinct trends with geochemical differentiation (i.e., with decreasing MgO: Supplementary Figure S1). The averages of the median values of the 7 intraoceanic arcs (Bismark-New Britain, Kurile, Kermadec, Mariana, New Hebrides, South Sandwich, Tonga) with thin crust thickness (< 20 km) display a systematic increase of Zn contents with decreasing MgO from an initial value of ~ 70 ppm up to 90–100 ppm Zn at MgO values between 1 and 2 wt%, after which Zn decreases to values of 40–50 ppm for the most evolved rocks (Fig. 1a and Supplementary Figure S1). However, several thin arcs do not show such a decrease because they do not evolve to rocks differentiated enough (Supplementary Figure S1).

Average values of zinc median contents of magmas emplaced in thick arcs (> 30 km: Mexico, Aleutians, Cascades, NVZ), in contrast, display flat to slightly declining trends with decreasing MgO down to MgO values ~ 1.5 wt%, after which Zn decreases along a steeper trend in the most differentiated rocks to an average value of ~ 40 ppm (Fig. 1a and Supplementary Figure S1). Zn trends in magmas of intermediate thickness arcs display an intermediate behavior (Supplementary Figure S1). The most differentiated magmas of the Lesser Antilles arc display an increase in Zn contents rather than a decrease like all other arcs (Supplementary Figure S1).

The trend of MOR magmatic rocks is characterized by a steeper negative slope than the average trend of the thin arcs in the Zn-MgO space (Fig. 1a). The most primitive MOR basalts have similar median Zn contents as the most primitive thin and thick arc basalts (~ 75 ppm) and grow to a median Zn content of ~ 170 ppm at ~ 2 wt% MgO, that is significantly higher than the average value of thin arcs (Fig. 1a), after which Zn contents drop to a median value of ~ 100 ppm with further differentiation.

In order to quantify the differences of the Zn–MgO trends in different arcs, slopes of the linear regressions between Zn and MgO were calculated for the early differentiation segments, i.e., excluding the median values corresponding to the strong Zn decreases (or increase for the Lesser Antilles) in the most differentiated rocks of all arcs (Table 1 and Supplementary Figure S1). Thicker arcs are characterized by positive Zn–MgO slopes, whereas thin arcs are characterized by negative Zn–MgO slopes and intermediate thickness arcs have intermediate slope values (Fig. 1b). Several arcs, which do not display statistically significant correlations between Zn and MgO (e.g., Bismark-New Britain, Ryukyu, Cascades, Luzon, Honshu, Central America: Table 1), are represented by shaded symbols in Fig. 1b. The slopes so calculated display a statistically significant correlation with the thickness of the corresponding arc crust (Fig. 1b).

**Table 1:** Summary of the values of the regression slopes (MgO vs. SiO_2_ and Zn vs. MgO) and associated statistical values.

Arc	Crust thickness (a)	Error (a)	Type according to crust thickness (b)	slope MgO–SiO_2_	Error (c)	r^2^ (c)	Slope Zn–MgO	Error (c)	r^2^ (c)	Average of median Fe_2_O_3tot_ at MgO 4–6 wt% (d)	Error (d)	Average of median Sr/Y at MgO 4–6 wt% (e)	Error (e)
S. Sandwich	11.8	0.1	< 20 km	− 1.7719	0.2444	0.913	− 5.0157	0.7201	0.802	10.6	0.3	7.3	0.7
Mariana	14.5	1	< 20 km	− 1.9778	0.2066	0.884	− 4.6166	0.52011	0.840	11	0.3	14.1	2.3
Kermadec	15	3	< 20 km	− 1.3980	0.3308	0.749	− 3.9468	0.6076	0.710	11	0.5	10.5	2.9
New Hebrid	15.6	0.2	< 20 km	− 1.6128	0.4708	0.516	− 2.1136	0.8971	0.270	11.2	0.9	29.6	3.8
Kuriles	18.3	0.9	< 20 km	− 1.5281	0.2560	0.798	− 2.0405	0.7744	0.332	9.2	0.2	15.3	0.9
Tonga	20	0	< 20 km	− 1.4867	0.2237	0.786	− 2.6703	0.6144	0.486	11.1	0.3	11.9	1.8
Bismark-NB	22.5	6.5	< 20 km	− 1.7614	0.2099	0.865	− ***0.7489***	***0.4575***	***0.124***	9.8	0.3	27.9	7.9
Ryukyu	24.5	3.4	20–30 km	− 1.2420	0.3479	0.614	***3.2332***	***1.4993***	***0.215***	9.6	1	14.6	1.4
Kamchatka	24.6	5.4	20–30 km	− 1.1359	0.3028	0.501	− ***0.7217***	***0.5025***	***0.108***	9.2	0.3	19.3	1.7
Lesser Antilles	24.7	0.7	20–30 km	− 1.0392	0.2039	0.743	1.4182	0.4301	0.420	9.3	0.2	16.3	4.8
Aeolian	24.9	1	20–30 km	− 0.9813	0.1454	0.820	2.2710	0.5283	0.627	8.5	0.1	30.8	2
Sulawesi	27.4	2.2	20–30 km	− 0.9439	0.4256	0.451	− 1.1683	0.5830	0.334	10.3	0.5	20.2	1.8
Luzon	27.8	4.5	20–30 km	− 1.0296	0.0941	0.916	− ***1.0374***	***1.5086***	***0.044***	9	0.5	25.9	3.4
Central America	28	7	20–30 km	− 0.8691	0.0470	0.961	− ***0.8611***	***0.4467***	***0.179***	9.6	0.3	31	2.8
Aegean	28.2	0.6	20–30 km	− 0.7484	0.1090	0.825	− 2.1850	0.5792	0.587	7.5	0.8	24	3.9
New Zealand	28.6	3.2	20–30 km	− 0.7093	0.1482	0.638	2.8065	0.5898	0.618	7.8	0.3	na	na
Honshu	29.2	2.6	> 30 km	− 1.4021	0.1133	0.922	− ***0.2739***	***0.3475***	***0.029***	9.7	0.3	na	na
Mexico	30.3	5.5	> 30 km	− 0.6281	0.0410	0.933	1.2997	0.3705	0.370	7.6	0.5	27.4	2
Aleutians	37.5	2.5	> 30 km	− 0.8012	0.0885	0.911	2.6022	0.5834	0.434	9.4	0.2	18.3	3.9
Cascades	38.8	1.9	> 30 km	− 0.9349	0.0950	0.898	***0.2498***	***0.4216***	***0.019***	8.4	0.5	30.3	1.6
NVZ	42	9.8	> 30 km	− 0.4906	0.0236	0.971	3.7100	0.6348	0.698	7.6	0.2	40.1	0.9
MOR	6.5	0.5	< 20 km	− 2.3322	0.3155	0.785	− 10.076	0.6704	0.922	14.3	1.7	4.1	1.5

Bold and italics indicate poor statistical correlations (see Supplementary Figure S1).

(a) From^64^, except Tonga thickness, which is from^65^, Kermadec thickness, which is from^66^, Aleutians thickness which is from^67^, and Northern Andes thickness which is from^68^. The Tonga crustal thickness here taken corresponds to the maximum crustal thickness of^65^ because arc magmatism occurs in coincidence with the thickest part of the Tonga arc (Fig. 9 in^65^). Crustal thicknesses have been calculated by^64^ using the global crustal model at 2° × 2°, CRUST 2.0, administered by the US Geological Survey and the Institute for Geophysics and Planetary Physics at the University of California^69^, which is an updated version of CRUST 5.1, a global crustal model at 5° × 5°^70^.The model is based on seismic refraction data published up to 1995 and a detailed compilation of sediment thickness. The crustal thicknesses of^64^ are within the ranges of crustal thicknesses reported in previous studies^71,72^ with which they show good linear correlations (r = 0.70 with respect to crustal thicknesses of^71^, and r = 0.74 with respect to crustal thicknesses of^72^). Oceanic crust thickness is from.

(b) Attribution to a crust thickness type takes into account the 1σ uncertainty and the geochemical systematics: for instance, Bismark/New Britain has average crust thickness slightly above 20 km, but taking into account the 1 uncertainty minimum values are largely < 20 km and geochemical trends are more typical of arcs < 20 km thick. This subdivision is purely semantic and does not change the mathematical correlations.

(c) errors and correlation coefficients r^2^ of the regressions were calculated using the "LINEST" function in Excel.

(d) From^7^.

(e) Fromy^8^.

**Figure 2:**
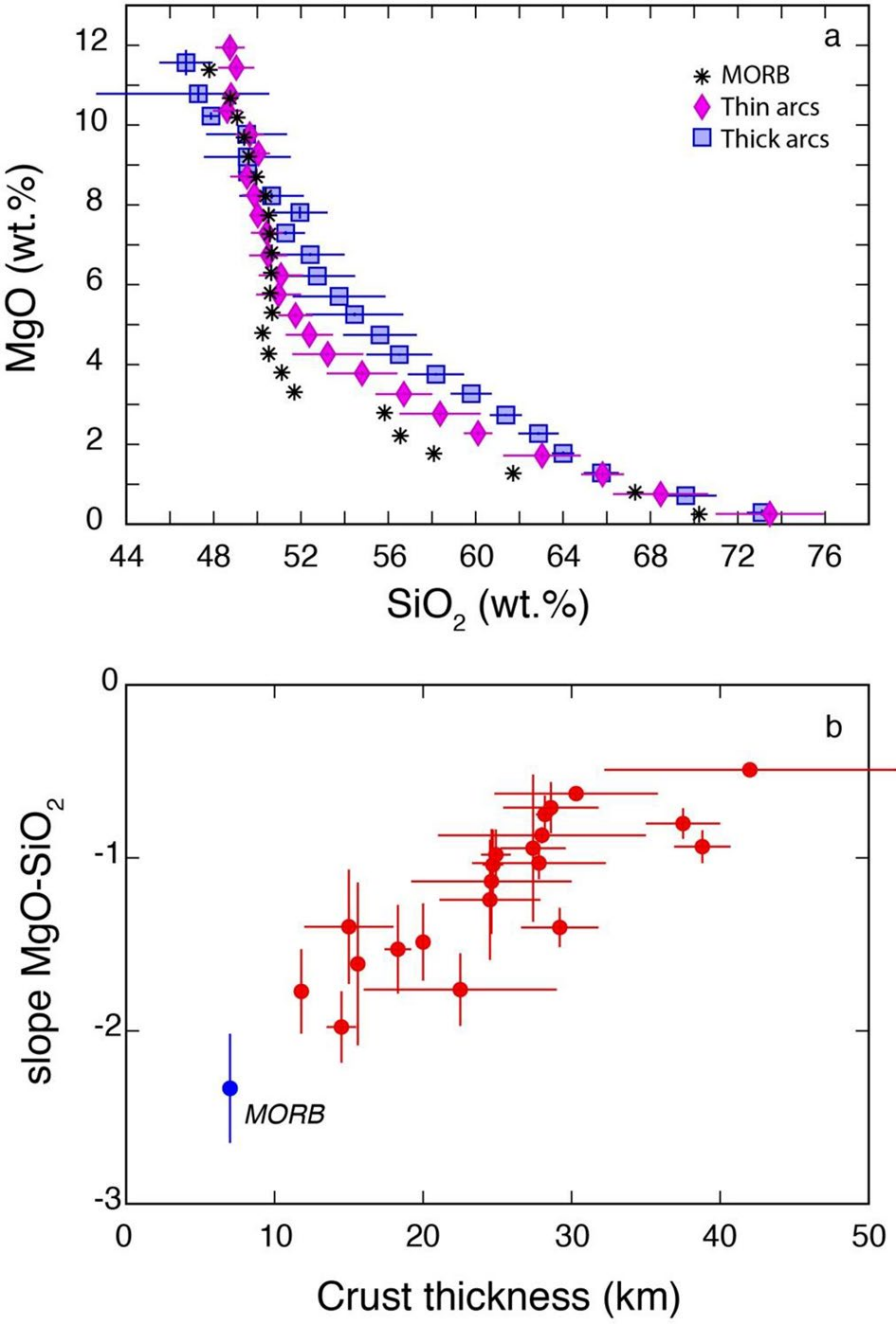
MgO-SiO_2_ systematics of arc and MOR magmatic rocks. (**a**) MgO-SiO_2_ plot of the averages of median values of all thin (< 20 km) and all thick (> 30 km) arcs compared to median values of MOR magmas for MgO bins ~ 0.5 wt% (data reported in Supplementary Data File 1); (**b**) plot of the MgO-SiO_2_ slopes of the early evolutionary parts (MgO > 2–5 wt% depending on the arc) of individual arcs and MOR magmatic rocks versus crustal thickness (data reported in Table 1).

Also MgO–SiO_2_ trends of the early evolutionary paths of arc magmas (i.e., before a break in the slope of the trend to a significantly shallower slope for MgO contents below a variable arc-dependent threshold of 2–5 wt%; Supplementary Figure S2) display different slopes, steeper in thin arcs and shallower in thick arcs (Fig. 2a, Supplementary Figure S2, and Table 1). MOR magmatic rocks display an overall steeper slope than thin arcs during early evolution in the MgO–SiO_2_ space (Fig. 2a). The values of the slopes defined by the early to intermediate differentiation trend of arc and MOR magmas in the MgO–SiO_2_ space display again significant correlations with the crust thickness of the corresponding arc and of MOR (Fig. 2b). Although there might be some degree of arbitrariness in the choice of the point at which the slope breaks in the MgO–SiO_2_ space (especially for some arcs: Supplementary Figure S2), changing the break point to higher MgO values does not significantly change the slope of arcs in such a way to affect the correlation of Fig. 2b. In fact, inter-arc changes of such slopes are much larger than the small changes that arise from a different choice of the break point within a specific arc.

### Crustal thickness control on fractionating mineral assemblages

The data reduction of the large dataset used in this study leads to results (median values) that statistically represent the most common values within a population with a dominantly normal distribution like that for the elements here considered within the ~ 0.5 wt% MgO bins. This means that the interpretation of these results is forcedly a simplification of the processes occurring in arcs, as indicated by the large distribution clouds of single rock analyses compared to the median values calculated from them for the MgO bins within each arc (Supplementary Figures S1-S2). Therefore, the processes interpreted on such a basis are first-order processes and do not exclude, within each specific arc, the occurrence of additional second or lower order processes (e.g., ref.^23^). Such processes might be important in some arcs, where correlations of median values of elements are not statistically significant (e.g., Fig. 1b and Table 1).

The different trends displayed by magmas of distinct arcs (and MOR) in the Zn–MgO and MgO–SiO_2_ spaces (Figs. 1, 2 and Supplementary Figures S1-S2) must be, to their greatest extent, the result of differentiation processes occurring within the crust because of the large SiO_2_ and MgO ranges that they encompass (Supplementary Figures S1-S2). A variety of studies agree in considering differentiation of arc magmas as the complex result of various processes, including fractional crystallization^24,25^, recharge/mixing^13,26,27^, partial melting^28^ and assimilation of host rocks^29^. Arguably, fractional crystallization (and to some extent partial melting, which is the opposite process of fractional crystallization) can be considered as the main process responsible for the large SiO_2_ and MgO variability observed in arc magmas^25^. Superimposed on this, assimilation, mixing and recharge are also universal processes occurring in arcs that tend to homogenize the signals of fractional crystallization^27^.

Figure 1 shows that Zn displays different degrees of increase or decrease during magmatic differentiation that depend on the thickness of the arc crust. Because primitive basalts have very similar values (70–80 ppm) both in arcs of different thickness and in MOR (Fig. 1a) and because the continental (72 ppm: ref.^30^) and oceanic crust (~ 75 ppm: Fig. 1a) Zn contents also fall within this same range, wholesale assimilation of crustal lithologies (either oceanic or continental) cannot explain alone the Zn systematics observed in arcs and MOR magmas. Partial melting of crustal lithologies producing SiO_2_-rich melts could be a significant process in arcs, especially at higher depths (and therefore in thicker arcs) because of thermal constraints^31^. Mixing of basalt with such SiO_2_-rich and either Zn-poor or Zn-rich partial melts would be needed to explain the decreasing and increasing trends of Zn with MgO in thick and thin arcs, respectively.

However, almost all arcs show a kink in both the Zn–MgO and MgO–SiO_2_ trends suggesting that mixing with a SiO_2_-rich and Zn-poor or Zn-rich crustal melts is not a viable explanation either. Mixing and assimilation certainly occur in arcs (there is ample isotopic, petrographic and mineral chemistry evidence for that) and is probably also a cause of the scatter of single rock analyses around the kinked trends in the Zn–MgO and MgO–SiO_2_ spaces (Supplementary Figures S1-S2). However, mixing and assimilation in the crust must involve end-member magmas whose Zn–MgO–SiO_2_ systematics are controlled by fractionating or restitic minerals. Therefore, although treating the different trends observed in arcs in the Zn–MgO–SiO_2_ space as the result of fractional crystallization is an approximation, these trends ultimately tell us what are the mineral phases that are involved in producing their different slopes through combined fractional crystallization, partial melting, assimilation and mixing processes.

**Figure 3:**
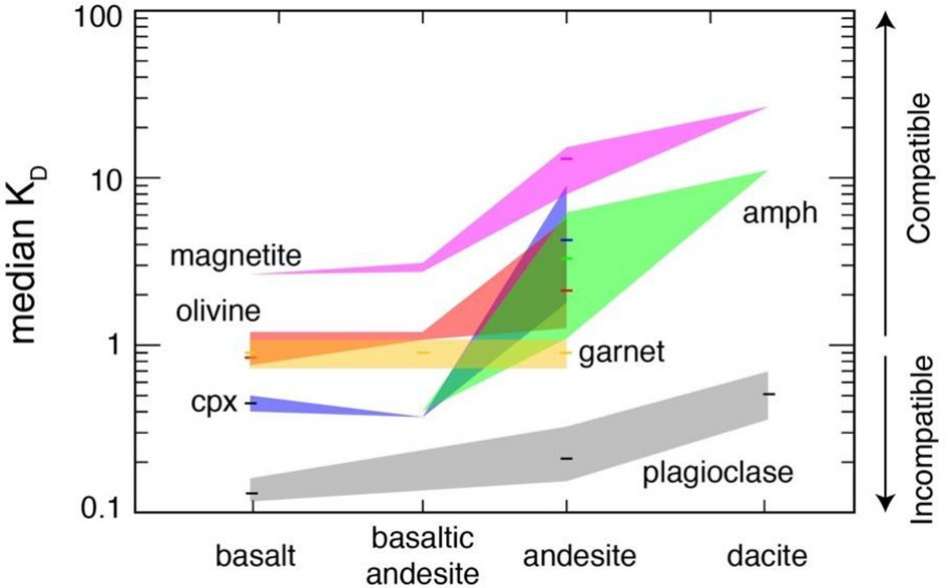
Zn partition coefficient (K_D_) values for mineral-melts of different compositions The K_D_ values are reported as median values (coloured ticks) and their 20th and 80th percentile (upper and lower boundaries of the coloured fields). Source data are reported in Supplementary Table S1.

Thus, for simplicity I will model the distinct Zn–MgO–SiO_2_ trends of arcs as the dominant result of fractional crystallization during which Zn behavior shifts from incompatible to compatible for magma differentiation occurring within an increasing crustal thickness (Fig. 1a). Such a behavior should be discussed considering the partition coefficients of Zn between melt and the main minerals fractionating in arc (and MOR) magmas (i.e., olivine, plagioclase, amphibole, clinopyroxene, garnet and magnetite). A compilation of K_D_ values from the literature (Supplementary Table S1 and Fig. 3) suggests that, among the potential fractionating phases during the early and intermediate stages of arc magma differentiation, magnetite is the one for which Zn has the highest affinity, compared to pyroxenes and particularly to plagioclase, which has very low K_D_ values for Zn. Zn is slightly incompatible in olivine in equilibrium with basalt but becomes compatible in this mineral when the latter crystallizes from basaltic andesite and andesite melt (Fig. 3). In contrast Zn is compatible in magnetite already crystallizing from basalt and its compatibility strongly increases with magmatic differentiation (Fig. 3). Zn has K_D_ values slightly < 1 for garnet in equilibrium with basaltic to andesitic melts^32^ (Fig. 3 and Supplementary Table S1). At intermediate stages of magmatic evolution (andesite, dacite) Zn becomes increasingly compatible in amphibole, clinopyroxene (Fig. 3) and biotite (K_D_ ~ 18 in dacitic melts: Supplementary Table S1). The onset, in the most evolved stages of arc magmas, of crystallization of biotite and magnetite, plus other accessory mineral phases (e.g., ilmenite) for which Zn has a strong affinity, is likely responsible for the strong Zn decreases in most arcs below 2–4 wt% MgO. Only some thin arcs do not display such a decrease (South Sandwich, Kermadec, New Hebrides, Kurile), perhaps because not enough differentiated rocks occur in the databases of these arcs. Zn contents display a strong increase in the most differentiated rocks of the Lesser Antilles arc. Although these trends in the most differentiated rocks may be of interest for those specific arcs, their discussion is beyond the scope of this work which considers the Zn-MgO trends of the early to intermediate differentiating magmas, excluding the most differentiated rocks.

### Modelling trends in the MgO–SiO_2_ and MgO–Zn spaces

In order to quantify the relationship of Zn–MgO–SiO_2_ systematics with fractionating mineral assemblage of arcs with different thickness, mass balance calculations using a Monte Carlo approach (see “Methods” and Supplementary Tables S2-S3) have been used to model simultaneously the Zn–MgO and MgO–SiO_2_ trends of the different arcs (and of MOR magmas) through fractionation of the main phenocrystic minerals occurring in mafic to intermediate arc and MOR magmas (olivine, amphibole, clinopyroxene, plagioclase, magnetite, garnet). This corresponds to reproducing the trends through fractionation of mineral assemblages in the tridimensional SiO_2_–MgO–Zn space. This approach provides stringent constraints to the combination of mineral proportions and residual melt fractions that satisfy simultaneously the Zn–MgO and MgO–SiO_2_ trends.

The model was run using a home-made RStudio script using the R software^33^ (see “Methods” and the examples of Supplementary Data File 1). The solutions of the simulations returned the combinations of bulk fractionating mineral assemblages and residual melt fractions able to reproduce the end points in the tridimensional SiO_2_–MgO–Zn space of the trends of each arc starting from appropriate parental compositions in the same tridimensional space (Supplementary Figures S1-S2).

**Figure 4:**
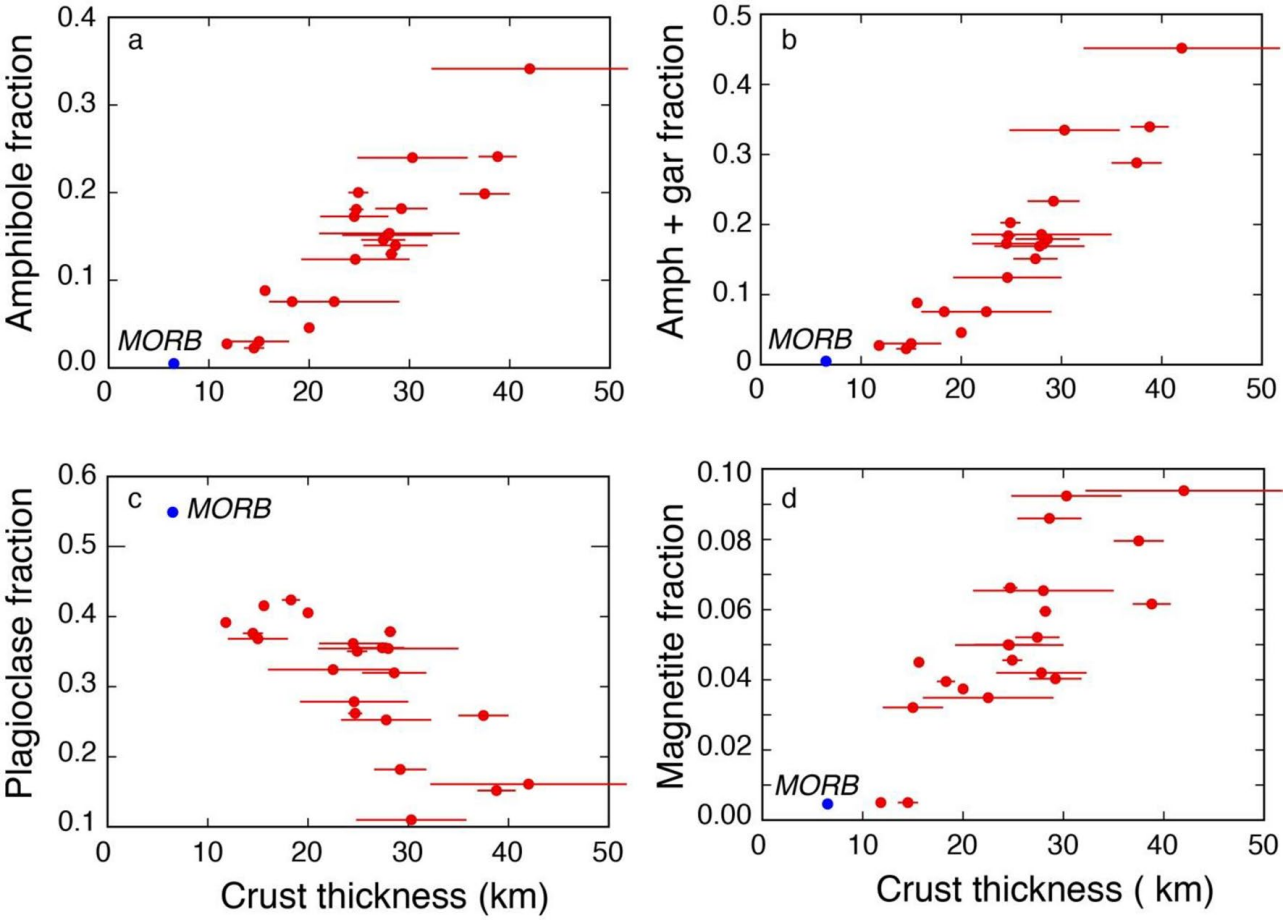
Results of Monte Carlo modelling of fractionating mineral proportions. (**a**) Proportion of amphibole fractionating in arc magmas with respect to arc thickness; (**b**) proportion of amphibole (Amph) + garnet (gar) fractionating in arc magmas with respect to arc thickness; (**c**) proportion of plagioclase fractionating in arc magmas with respect to arc thickness; (**d**) proportion of magnetite fractionating in arc magmas with respect to arc thickness. Data are reported in Supplementary Table S4.

Overall, results of the Monte Carlo simulations of fractionation processes applied to Zn–MgO–SiO_2_ systematics (Supplementary Table S4) show that the fractionating mineral assemblages gradually shift from plagioclase-dominated in thin arcs to amphibole-, magnetite-, and garnet-dominated in increasingly thicker arcs (Fig. 4). Clinopyroxene and olivine do not significantly correlate with crustal thickness, suggesting that these minerals act as buffers in the fractionating assemblages. High proportions of both olivine and plagioclase in fractionating magmas of thin arcs are needed to explain on one hand the steep decrease of MgO at low SiO_2_ values (olivine effect, because of SiO_2_/MgO < 1 in olivine: Supplementary Table S5) and on the other the broadly incompatible behavior of Zn (plagioclase effect, due to the very low K_D_ values of Zn in plagioclase: Fig. 3). In contrast, the high proportions of clinopyroxene, amphibole and garnet and the low proportions of plagioclase in fractionating magmas in thick arcs are consistent with both the shallower decrease of MgO with SiO_2_ (due to the high SiO_2_/MgO values in all these minerals, between 3 and 4: Supplementary Table S5) and with the slightly compatible behavior of Zn (due to the K_D_ values of Zn in these minerals around or slightly > 1: Fig. 3).

These results agree with petrographic observations that phenocrysts in relatively primitive thin arc rocks (e.g., Mariana, South Sandwich: refs.^23,34,35^) consist of olivine, plagioclase and pyroxenes (with virtually no amphibole), whereas relatively primitive rocks and cumulates of thick arcs (e.g., Ecuador, Mexico, Cascades, Central Andes: refs.^36–39^) contain variable amounts of amphibole. They also support and quantify the suggestion that extensive cryptic amphibole (and garnet) fractionation may occur in arcs^40^, especially in increasingly thicker ones.

### Crust thickness control on fractionating assemblages

The systematic correlations of the changing proportions of fractionating amphibole, garnet, magnetite, olivine and plagioclase with changing crustal thickness are consistent with experimental petrology results carried out on hydrous basaltic to andesitic melts fractionating at different pressures^41–48^. These results show that plagioclase, clinopyroxene and olivine are the main minerals crystallizing at the liquidus of hydrous mafic melts at low pressures (e.g., < 0.1–0.3 GPa depending on H_2_O content) whereas amphibole, garnet, clinopyroxene, and magnetite crystallize at or near the liquidus of hydrous mafic melts at high pressures (> 0.8 GPa). The data presented and discussed here suggest that there is a gradual and continuous crustal thickness-controlled change in the proportions of fractionating minerals between the above two end-member assemblages that results in the systematic changes of SiO_2_–MgO–Zn trends of arc magmas.

The preferential fractionation of amphibole and garnet in thicker arcs is unlikely to result only from higher H_2_O contents in the primitive basalts of thicker arcs^10,11,14^. In fact, available data point to similarly variable H_2_O contents of primitive arc basalts, comprised between 0.5 and 7wt%^49,50^, independent of their thickness^49^. A similar average H_2_O content (3.9 ± 0.4 wt%, 1SD) for primitive arc basalts has been suggested based on H_2_O contents measured in melt inclusions in olivine and other mafic minerals^49^, although such contents may be questioned because of post-entrapment hydrogen loss or gain^51^. Regardless, the occurrence of variable amounts of garnet required for the thicker arcs by the modelling here presented indicates pressures of crystallization of at least 0.8 GPa, even in H_2_O-rich magmas^43^. A thicker crust will result, as suggested by the data above discussed, in an average magma evolution at deeper levels^8^ which will increase H_2_O contents in the residual magma more significantly than magma evolution at shallower levels already in the early fractionation stages (Supplementary Note 2 in Supplementary Information), because of the strong pressure dependency of H_2_O solubility in silicate melts^52^. This will, in turn, further stabilize the fractionation of amphibole and garnet from relatively unevolved basaltic andesite and andesite magmas at mid- to lower crustal levels^42,43^. If a systematic H_2_O enrichment in primary basalts of thick arcs does occur^11,14^, this would further enhance amphibole and garnet fractionation in thick arcs.

**Figure 5:**
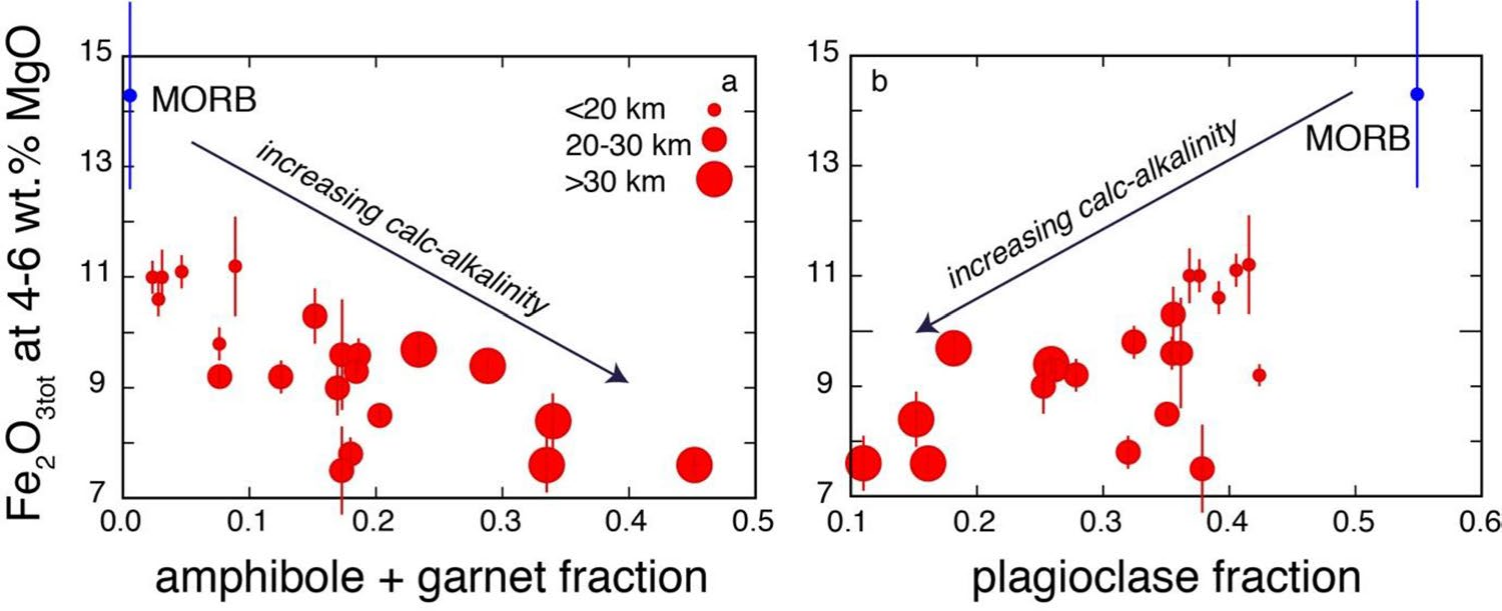
Proportions of mineral fractions versus tholeiitic character. (**a**) Proportions of fractionating amphibole + garnet versus the tholeiitic index (as defined by^7^); (**b**) proportions of fractionating plagioclase versus the tholeiitic index (as defined by^7^). Different circle sizes highlight different arc crust thicknesses.

The variable crustal thickness-controlled proportions of the fractionating mineral assemblages obtained by modelling Zn–MgO–SiO_2_ arc systematics also correlate with the median Fe_2_O_3tot_ values at 4–6 wt% MgO of arcs (Fig. 5), which are a measure of the tholeiitic versus calc-alkaline character of arc magmas^7,53^. Overall, these data suggest that first order processes of differentiation observed in arc magmas and the generation of a continuous transition from tholeiitic to calc-alkaline character are the result of pressure-dependent stability of different fractionating mineral phases in hydrous magmas (see also ref.^25^), which is ultimately controlled by crustal thickness. A thicker crust results in an average evolution of arc magmas at deeper crustal levels^8,54^ and, therefore, is characterized by fractionation of higher-pressure assemblages (olivine, clinopyroxene, amphibole, garnet, magnetite) from the hydrous basalts typical of the arc environment. This leads to the development of a typical calc-alkaline trend in associated arc magmas (Fig. 5). In contrast, a thinner crust results in an average shallower crustal evolution of arc magmas characterized by the fractionation of the assemblage olivine, plagioclase, pyroxenes from such hydrous basalts. This leads to the development of dominantly tholeiitic trends in associated arc magmas (Fig. 5). It is important to highlight that the results presented here suggest that there is a continuum between these two extremes without a neat subdivision between calc-alkaline and tholeiitic trends, but rather a gradual transition, that is controlled by the role that crustal thickness of the arc has on the proportions of fractionating minerals. This is consistent with the development of calc-alkaline trends also at intermediate to relatively low pressures under H_2_O-saturated conditions^44^. H_2_O contents on the high side of the 0.5–7 wt% range of primitive arc basalts, as discussed above, may thus result in subordinate calc-alkaline trends even in thin arcs otherwise dominated by tholeiitic trends^35^.

On the other hand, it is significant that MOR magmas, which are almost anhydrous^55^, fall on the continuation of the trends of Figs. 1b and 2b suggesting that Zn–MgO and MgO–SiO_2_ systematics seem to be rather insensitive to the largely different H_2_O contents of primitive basalts in MOR (~ 0.1–0.2 wt%: ref.^55^) and thin arcs (~ 0.5–7 wt%^49,50^) and that crustal thickness seems to be the main controlling factor on the different Zn–MgO–SiO_2_ systematics of magmas in these distinct settings.

## Discussion

The results above discussed have several implications for large-scale processes associated with arc magmatism, encompassing Zn contents in the mantle, the crust role in modulating the H_2_O flux from mantle to Earth surface, and the formation of porphyry Cu deposits.

**Figure 6:**
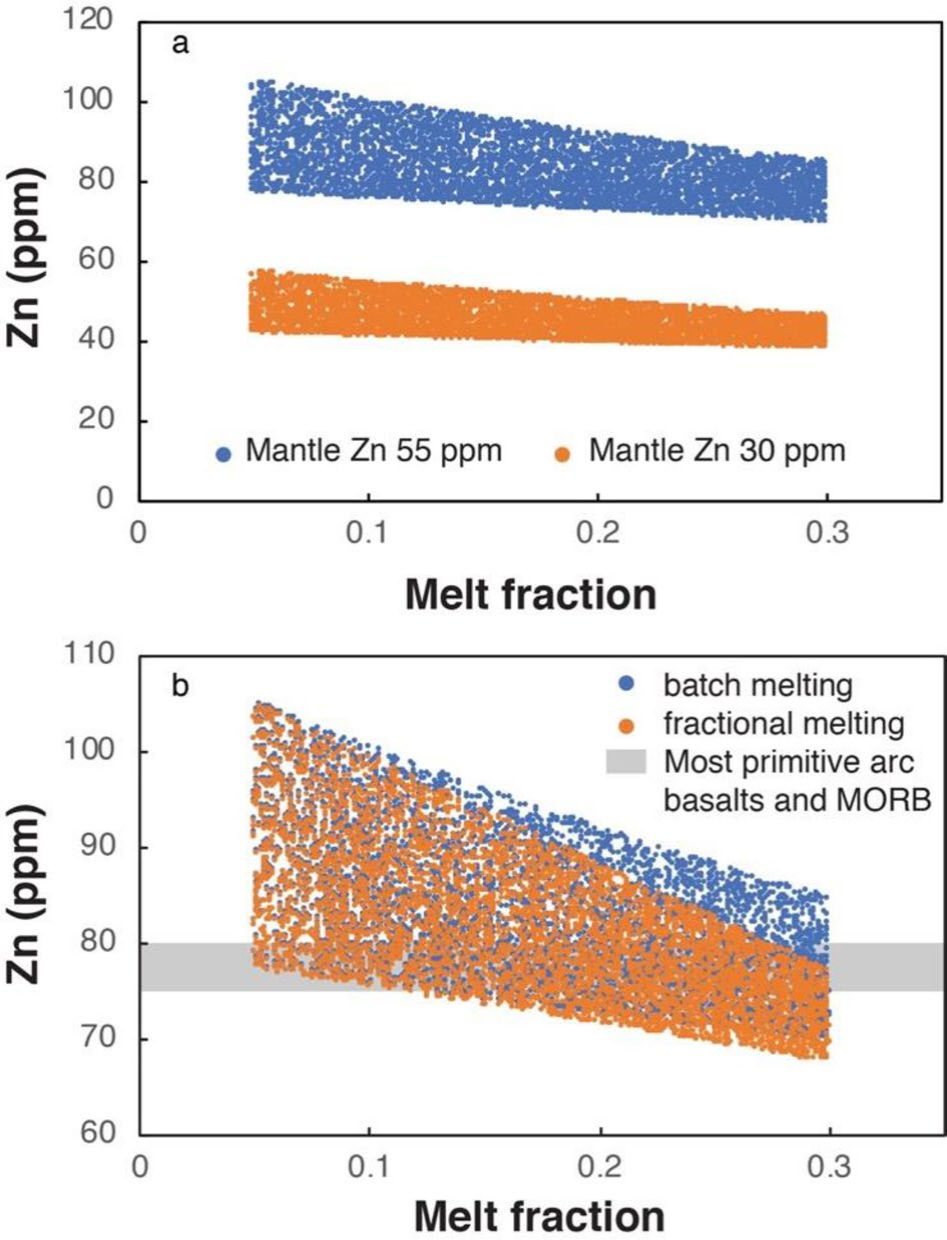
Zn contents of mantle-derived melts. (**a**) Zn contents of batch melting mantle-derived melts starting from mantles with different initial Zn contents (30 and 55 ppm); (**b**) Zn contents of batch melting and fractional melting mantle-derived melts starting from a mantle with 55 ppm Zn.

The most primitive basalts from both thick and thin arcs and from MOR have similar Zn contents (~ 75–80 ppm: Fig. 1a). This suggests that, like Cu^56^, arc basalts and MORB derive from a similar mantle in terms of Zn contents and that Zn, like Cu^57^, is not significantly enriched in the mantle wedge by subduction-related processes. A possibility allowing for a Zn flux from the subducted slab would be that the mantle wedge be depleted in Zn compared to MOR mantle (Fig. 6a). However, this seems to be inconsistent with available data suggesting similar Zn contents for both Primitive and Depleted Mantle (~ 55 ppm: https://earthorg/GERM) and even higher Zn contents in Enriched Mantle types (> 100 ppm^17^). Alternatively, differential partial melting in subduction versus MOR settings (“Methods” section) could possibly be compensated by a Zn flux from the slab in the subduction setting if mantle wedge-derived basalts were Zn-depleted with respect to MORBs. However, also this seems to be unlikely. In fact, using available partition coefficients for olivine, clinopyroxene and orthopyroxene^17^, that allow calculating the bulk partition coefficient between basaltic melt and the above mentioned lherzolite minerals, it results that Zn is only slightly incompatible during mantle melting (see “Methods” section). It follows that the 75–80 ppm content in a primary mantle-derived basalt is quite insensitive to the melt fraction, using either batch melt or fractional melt models (Fig. 6b). In contrast to the similar Zn contents of primitive basaltic rocks in arcs and MOR, primitive Hawaii basalts display a significant Zn enrichment (> 100 ppm), which is consistent with their derivation from an enriched mantle source^22,58^ compared to that sourcing basalts in arcs and MOR (Fig. 1a).

**Figure 7:**
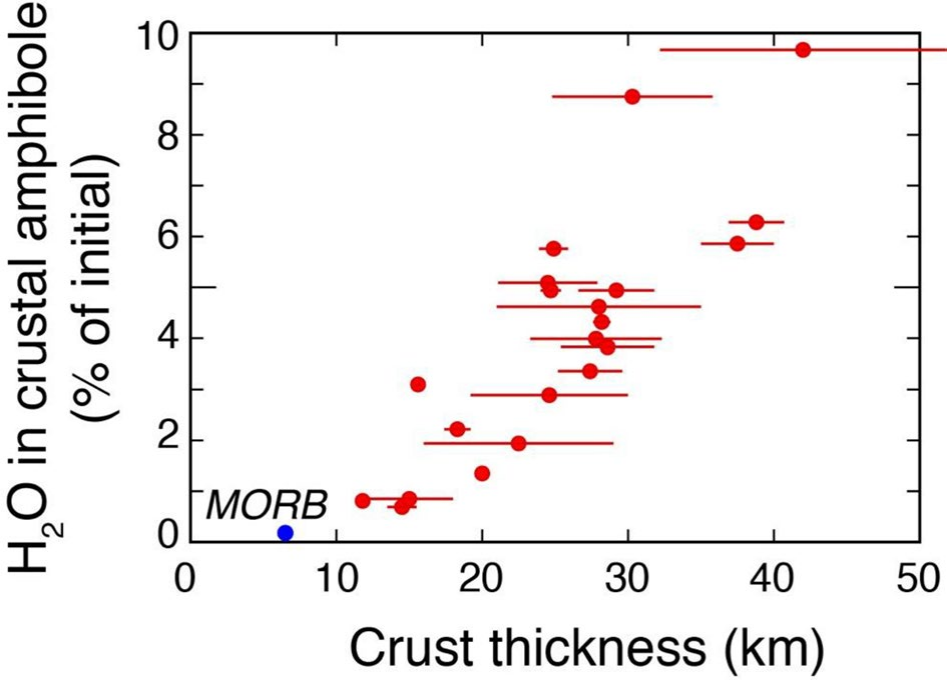
H_2_O stored in amphibole crystallized within the crust versus crustal thickness. H_2_O is the percentage of the initial H_2_O (~ 4 wt%^49^) in primitive arc basalts. Data from Supplementary Table S5.

**Figure 8:**
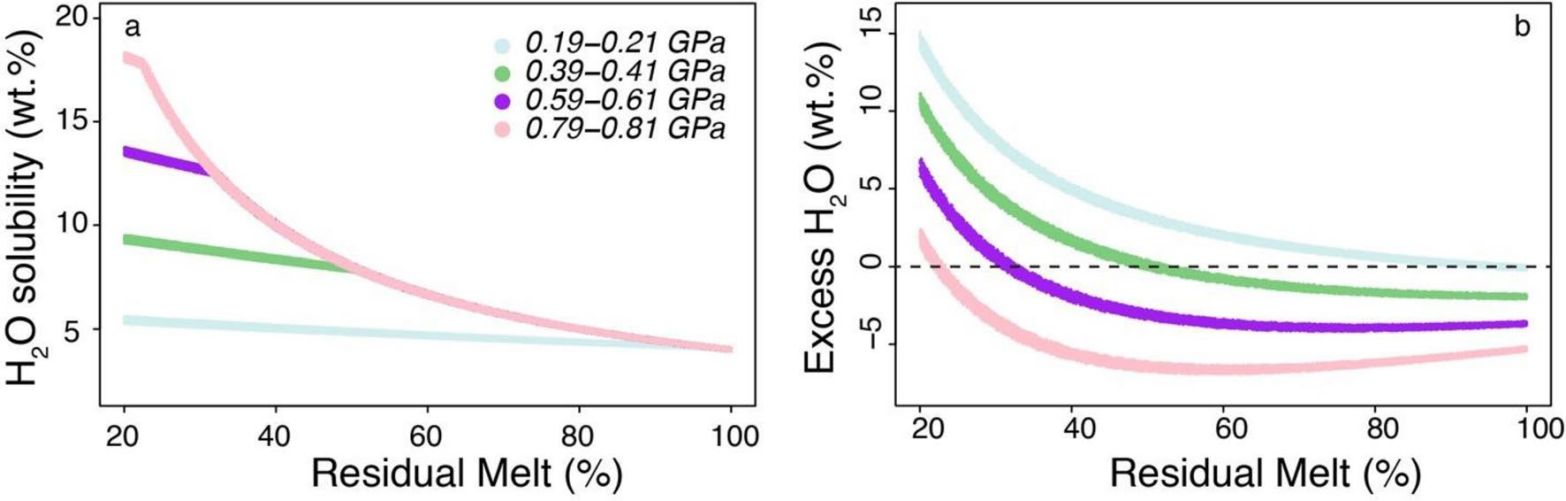
Variations of H_2_O solubility (**a**) and excess H_2_O (**b**) with changing residual melt fraction at 4 different pressures of magma fractionation (see Supplementary Note 2 in Supplementary Information). The starting H_2_O content at all pressures is 3.9–4.1 wt% and the H_2_O is considered to behave as completely incompatible. The pressure and melt composition dependency of H_2_O solubility in silicate melts are from the parametrization of ref.^59^ based on the model of ref.^52^. The different curves are the result of > 1000 simulations for ranges of initial H_2_O content of 3.9–4.1 wt% and pressures ranges of 0.19–0.21, 0.39–0.41, 0.59–0.61 and 0.79–0.81 GPa. The kinks of the curves in (**a**) indicate the point at which H2O saturation occurs (i.e., when on the right hand plot the curves raise above 0 wt% excess H_2_O). In Fig. 8b negative values indicate H_2_O-undersaturated conditions whereas positive ones indicate H_2_O-saturated conditions. At 0.2 GPa the magma is already saturated without any crystallization if it has an initial H_2_O content between 3.9 and 4.1 wt%. The plot was drawn using the RStudio interface of the R software^33^.

The results of this work also indicate that the suggested role of amphibole as a crustal filter of mantle-derived water is modulated by the crustal thickness of the arc: in other words, not all arcs act as a crustal sponge for H_2_O. In fact, results here presented suggest that intermediate magmas in the thinnest arcs carry towards the surface > 99% of the initial H_2_O content (e.g., average ~ 4 wt%^49^) of their basaltic parent (because very little H_2_O is lost to the trivial amounts of amphibole crystallizing in thin crust: Fig. 7 and Supplementary Table S5). This corresponds to theoretical H_2_O concentrations of such intermediate magmas of ~ 10 wt% (assuming that they correspond to ~ 0.4 residual melt fraction as suggested by the model here presented: Supplementary Table S5). In order to solubilize this H_2_O concentration in an intermediate silicate melt a depth of at least ~ 20 km (~ 0.6 GPa) is needed^52^ (Fig. 8), which is thicker than that of the thinnest intraoceanic arcs (Table 1). Therefore, magmas in such thin arcs exsolve and loose water since the early stages of differentiation because they cannot evolve at depths high enough to allow them to retain in solution the H_2_O they carry from the mantle during differentiation (Fig. 8; Supplementary Note 2 in Supplementary Information). Thus, the mantle-derived H_2_O is nearly completely fluxed towards the surface in thin arcs (Fig. 8).

In contrast, primitive basalts in thick continental arcs can lose up to ~ 10% of their initial H_2_O (e.g., ~ 4 wt%^49^) to amphibole crystallized in the crust (Fig. 7 and Supplementary Table S5). This implies the formation of progressively more abundant amphibole-rich cumulates in the crust of increasingly thicker arcs. Therefore, only in thick arcs a significant portion of mantle-derived H_2_O is, at least temporarily, locked in amphibole-rich cumulates. Such structurally-bound H_2_O may subsequently be released during ascent of magma incorporating cumulate amphibole^39^ or during metamorphism to promote dehydration-assisted crustal melting or source fluids that may be at the origin of different types of ore deposits^60^.

**Figure 9:**
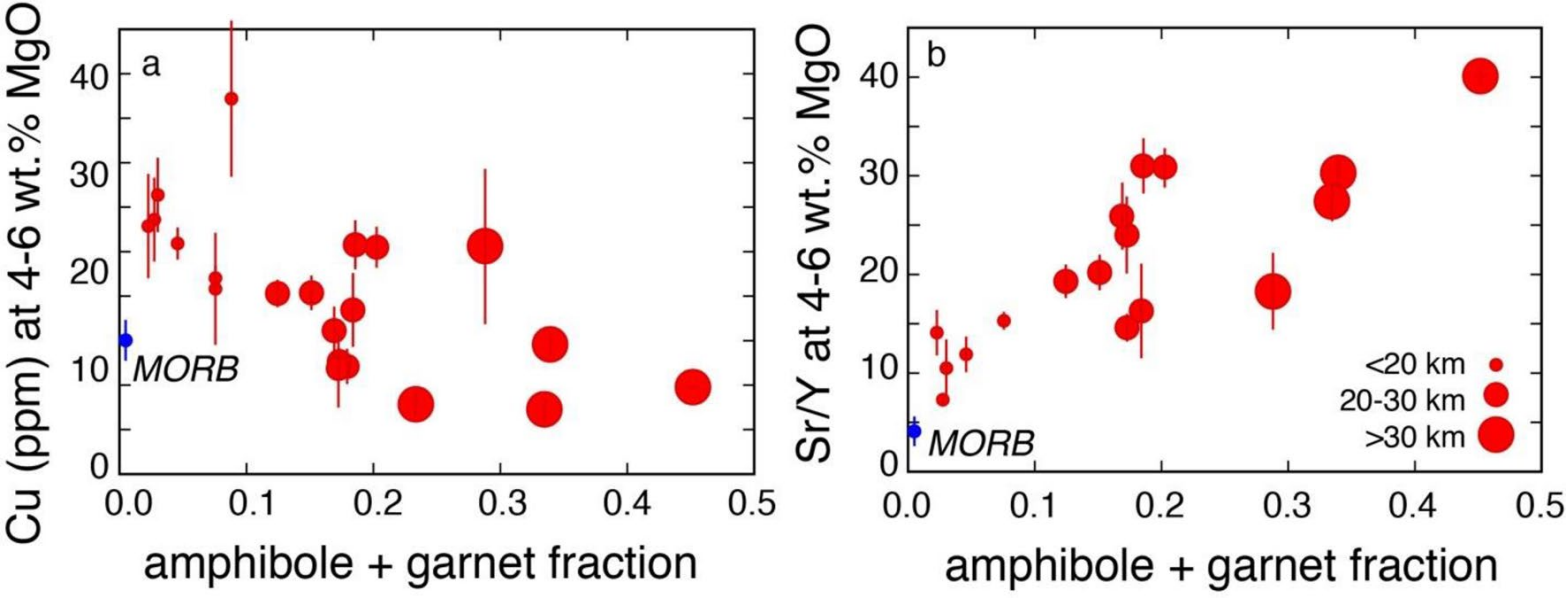
Proportions of fractionating minerals versus Cu and Sr/Y. (**a**) Proportions of fractionating amphibole versus Cu at 4–6 wt% MgO (as defined by^7^); (**a**) proportions of fractionating plagioclase versus Sr/Y at 4–6 wt% MgO (as defined by^8^). Different circle sizes highlight different arc crust thicknesses. Bismark-New Britain and New Hebrides are excluded from the plot b because their high Sr/Y values likely result from slab melting^8^.

The data here presented and discussed also provide quantitative explanations for the link between various geochemical systematics of arc magmas (e.g., Cu and Sr/Y^7–9^) and arc thickness-controlled fractionation of different mineral assemblages. The systematic loss of Cu in intermediate magmas of increasingly thicker arcs could be the result of continuous iron depletion in this setting^61^ due to fractionation not only of magnetite^7^ and garnet^61^, as previously suggested, but also of abundant amphibole (Figs. 5 and 9a) as recently suggested by ref.^62^: fractionation of all these minerals drive the evolution of thick arc magmas into the calc-alkaline field. A dominant proportion of amphibole in the fractionating assemblage of thick arc magmas (Fig. 4a,b) is also consistent with the dominant spoon-shaped REE patterns of thick arc magmas^62^. Also the progressively higher Sr/Y values in intermediate magmas of increasingly thick arcs^8,9^ can be quantitatively explained by the increasing amounts of fractionating amphibole plus garnet (Fig. 9b) and decreasing amounts of fractionating plagioclase in thicker arcs. All these inter-correlations suggest an overarching role played by crustal thickness-controlled differential fractionation of amphibole plus garnet, plagioclase and magnetite in the development of variable Sr/Y and calc-alkaline signatures in arc magmas. As already pointed out by ref.^8^, high Sr/Y magmas display the strongest calc-alkaline affinities (i.e., lowest Fe_2_O_3tot_ values at MgO contents between 4 and 6 wt%).

Despite the apparent negative effects of the thick arc setting on H_2_O and Cu contents of residual thick arc magmas discussed above, the latter are the most fertile for the formation of porphyry Cu deposits because they compensate such losses with a deeper crust evolution of the magmas^59^. This leads to significant magma volume accumulation through time in the lower to mid-crust, with such magma being characterized by high H_2_O concentrations^59^ because of the pressure dependency of H_2_O solubility in silicate magmas^52^ (Fig. 8; Supplementary Note 2 in Supplementary Information). In fact, in the thickest arc settings, residual intermediate magmas, despite losing ~ 10% of the initial H_2_O to amphibole crystallizing in the crust (Fig. 7), still hold ~ 90% of the average initial mantle-derived (e.g., ~ 4 wt%) H_2_O content^49^. This corresponds to H_2_O concentrations between ~ 7 and ~ 11wt% in the residual melts (for residual melt fractions between 30 and 50% as calculated in the model here presented: Supplementary Table S5 and Fig. 8). These H_2_O contents are soluble in intermediate melts at depths between 12 and 22 km^52^ which are largely within the crustal thickness of thick arcs. Such large amounts of H_2_O can be subsequently released when thick arc intermediate magmas ascend to shallower levels where they can form porphyry Cu deposits^59^. These data could finally explain the association of Au-rich porphyry Cu–Au deposits with thinner island arcs (e.g., Indonesia, Papua New Guinea, Philippines: ref.^4^) which would be favored by the early H_2_O exsolution typical of thin arc magmas (Fig. 8). Such early fluid exsolution, during the magma differentiation process, allows Au partitioning into the fluid phase before magmatic sulfide saturation, which would strongly deplete Au in the residual magma and make Au-rich porphyry mineralization impossible^63^.

## Methods

### Arc crustal thickness

Average crustal thicknesses of arcs and associated uncertainties were taken from Zellmer (2008), except the thickness of Tonga^65^, Kermadec^66^, Aleutians^67^, and Northern Andes^68^. The Tonga crustal thickness here taken corresponds to the maximum crustal thickness of ref.^65^ because arc magmatism occurs in coincidence with the thickest part of the Tonga arc (Figure 9 in ref.^65^). Crustal thicknesses have been calculated by ref.^64^ using the global crustal model at 2° × 2°, CRUST 2.0, administered by the US Geological Survey and the Institute for Geophysics and Planetary Physics at the University of California^69^, which is an updated version of CRUST 5.1, a global crustal model at 5° × 5°^70^.The model is based on seismic refraction data published up to 1995 and a detailed compilation of sediment thickness. The crustal thicknesses of ref.^64^ are within the ranges of crustal thicknesses reported in previous studies^71,72^ with which they show good linear correlations (r = 0.70 with respect to crustal thicknesses of ref.^71^, and r = 0.74 with respect to crustal thicknesses of ref.^72^). Oceanic crust thickness is from ref.^73^.

### Data collection and treatment

Geochemical data of bulk volcanic rocks (N = 42,600) from 21 recent arcs were collected from the Georoc database (http://georoc.mpch-mainz.gwdg.de/georoc/; Supplementary Data File 1). The Central Volcanic Zone of the Andes was excluded because of the very large thickness of the crust that biases the correlations between geochemical parameters and arc thickness (see also ref.^10^). The Izu Bonin arc was excluded because of the extensive occurrence of boninitic rocks that are formed through different processes than typical arc basalts in the other arcs. As in ref.^7^, to reduce the bias induced by outliers and to extract information on general trends, median values of Zn, SiO_2_, and MgO for subpopulations corresponding to bins of 0.5 wt% MgO were calculated (Supplementary Data File 1). When less than 10 data were available for one of the investigated elements within the 0.5 wt% MgO bin, the MgO interval was extended to a higher value (e.g., 1 or 1.5 wt%) to incorporate more values of that element. For comparison with arc systematics, data were also collected for MORB (https://www.earthchem.org/; N ~ 24,000) and for Hawaii (http://georoc.mpch-mainz.gwdg.de/georoc/; N ~ 5000), and treated in the same way as for arcs.

Linear correlations for the median arc values in the Zn–MgO and MgO–SiO_2_ spaces were detected for the arc products going from the most primitive rocks to variably evolved rocks, depending on the arc (Supplementary Figures S1-2). Statistically significant linear correlations in the Zn–MgO space were detected down to variably low MgO values ~ 0–4 wt% (Supplementary Figure S1 and Table 1), after which, in most arcs, Zn drops to low values. Statistically non-significant correlations are highlighted in Table 1.

The linear correlations considered in the MgO–SiO_2_ space were those regarding the early to intermediate stages of magmatic evolution (e.g., for MgO > 2–5 wt%). Below MgO values in the 2–5 wt% range, depending on the arc, the more differentiated median values displayed a visible change in the slope with respect to the less evolved median values (Supplementary Figure S2).

### Modelling the linear trends in the tridimensional Zn–MgO–SiO_2_ space by fractional crystallization

The linear Zn–MgO and MgO–SiO_2_ trends were modelled in the Zn–MgO–SiO_2_ space for a pure fractional crystallization process using simple mass balance equations (Supplementary Note 1 in Supplementary Information) in which the starting composition of the parental melt (Supplementary Table S2) was changed by subtracting variable proportions of 6 typical fractionating minerals in arc and MOR magmas (olivine, clinopyroxene, amphibole, plagioclase, magnetite, garnet). The objective of the model was to constrain the statistically most probable fractionating mineral assemblage (in terms of mineral proportions) and the extent of fractionation (residual melt fraction).

For each arc the composition of the most primitive median value was taken as that of the parental melt (Supplementary Table S2 and Supplementary Figures S1-S2). Sometimes, when the median values showed some scatter on the parent side, the parent compositions were allowed to vary within a tight range (< ± 0.2 wt% for SiO_2_ and MgO, ≤ 4 ppm for Zn; Supplementary Table S2). The target values were the compositions of the most evolved median values (i.e., the points with the lowest MgO content) of the individual linear MgO-SiO_2_ and Zn–MgO trends, i.e., the last points of these trends on the low MgO side before the change in the slopes (Supplementary Figure S2; target compositions in Supplementary Table S2). The target compositions were also allowed to vary within <  ± 0.2 wt% for SiO_2_ and MgO and ≤ 4 ppm for Zn (Supplementary Table S2). The model was run using a Monte Carlo approach, i.e., besides the small ranges of parent and target compositions discussed above, also the mineral compositions were allowed to vary randomly within given ranges (Supplementary Tables S3-S4). MgO and SiO_2_ mineral composition ranges were derived from experimental petrology and were typical of those minerals fractionating in basaltic to andesitic magmas (Supplementary Table S3). The Zn contents in those same minerals were instead modelled using a range of Zn K_D_ values for those minerals crystallizing from basaltic to andesitic magmas (Supplementary Tables S1 and S3).

The model was initially run (5 million simulations) leaving a large degree of freedom for the proportions of all potential fractionating minerals (i.e., 0–0.7 for plagioclase, amphibole, clinopyroxene, olivine, 0–0.3 for garnet and 0–0.15 for magnetite) and for the residual melt fraction (20–70%). The 5 million simulation runs were carried out for these broad ranges between 15 and 20 times for each arc to identify the most recurrent proportions of minerals and residual melt fractions. After this initial step, the ranges of fractionating minerals were narrowed down progressively to narrower ranges around the median values obtained in step 1 until a variably high number of successful solutions were obtained (depending on the arc), most of the times with a normal distribution (i.e., average ~ mean value: Supplementary Table S2) of all parameters involved (mineral proportions, residual melt fraction). Then, 5 million simulation runs, corresponding to the conditions (mineral proportions and residual melt fraction ranges) returning the highest number of solutions, were repeated five times for each arc and an average value with associated standard deviation was calculated from those 5 repeats (Supplementary Table S4; Supplementary Data File 2).

It is important to highlight that the model was run in order to obey simultaneously to the constraints imposed by both the Zn–MgO and MgO–SiO_2_ trends (in a tridimensional MgO–SiO_2_–Zn space). This reduces significantly the number of successful solutions but at the same time provides more stringent constraints on mineral proportions and residual melt fractions.

### Modelling of Zn contents in basaltic melts from partial mantle melting

The Zn content of melt from mantle melting was calculated for both batch and fractional melting (Fig. 6b) using the following equations, respectively:

1 $${Zn}_{melt}=\frac{{Zn}_{mantle}}{\left[{K}_{D-bulk}\left(1-F\right)+F\right]}$$

2 $${Zn}_{melt}=\frac{{Zn}_{mantle}}{{K}_{D-bulk}}{\left(1-F\right)}^{1/\left({K}_{D-bulk}-1\right)}$$

where ${Zn}_{melt}$ is the Zn concentration in the melt (in ppm), ${Zn}_{mantle}$ is the Zn concentration of the mantle (55 ppm or 30 ppm in the calculations), ${K}_{D-bulk}$ is the bulk partition coefficient for Zn between mantle lherzolite and melt, and *F* is the melt fraction.

More than 5000 simulations were run using the above equations and allowing random variations of *F* between 0.05 and 0.3 and of ${K}_{D-bulk}$ between 0.5 and 0.7. The range of *F* encompasses potential melt fractions in the sub-arc and sub-MOR mantle^74^ whereas the range of ${K}_{D-bulk}$ is a common range of values based on olivine, clinopyroxene and orthopyroxene partition coefficients between melt and peridotite^17^ and typical proportions of these minerals melting from a lherzolite.

### Modelling the H_2_O solubility of silicate melt during fractional crystallization at different pressures

Variations of H_2_O solubility and excess H_2_O with changing residual melt fraction (100 to 20%) at 4 different pressures (0.2, 0.4, 0.6, 0.8 GPa) of magma fractionation were modelled from the parametrization of ref.^59^ based on the model of ref.^52^ (see Supplementary Note 2 in Supplementary Information). The different curves are the result of 500,000 simulations for ranges of initial H_2_O content of 3.9–4.1 wt% and pressures ranges of 0.19–0.21, 0.39–0.41, 0.59–0.61 and 0.79–0.81 GPa. The H_2_O is considered to behave as completely incompatible in the model.

## Supplementary Information

Supplementary Information.

## Data Availability

All data needed to evaluate the conclusions in the paper are present in the paper and/or in the Supplementary Information.
