# Supplementary material for: Zinc systematics quantify crustal thickness control on fractionating assemblages of arc magmas
Source: Sci Rep. 2021 Jul 19;11:14667. doi: 10.1038/s41598-021-94290-6 (PMC8289985; doi:10.1038/s41598-021-94290-6)
Supplement: Supplementary file 1 — Supplementary Information. [file 41598_2021_94290_MOESM1_ESM.pdf]

Supplementary Information

Zinc systematics quantify crustal thickness control on fractionating assemblages of arc magmas

M. Chiaradia[1]

[1] Department of Earth Sciences, University of Geneva, Rue des Maraîchers 13, 1205-Geneva, Switzerland, e-mail: Massimo.Chiaradia@unige.ch

- Fig. S1. Zn-MgO plots of MORB and 21 arcs.
- Fig. S2. MgO-SiO_2 plots of MORB and 21 arcs.
- Table S1. Partition coefficients for Zn between mineral and silicate melts of different compositions.
- Table S2. Input values used for the Monte Carlo modelling.
- Table S3. Ranges of Zn K_D values for mineral-melt used for Monte Carlo modelling.
- Table S4. Proportions of fractionating mineral assemblages from Monte Carlo modelling.
- Table S5. Calculations to determine the amounts of H_2O stored in crystallized cumulate of arcs.
- Supplementary Note 1: RStudio Code for Monte Carlo simulations of the Zn-MgO and MgO- SiO_2 correlations.
- Supplementary Note 2: RStudio Code for Monte Carlo simulations of H2O solubility in fractionating magmas.

Figure S1: Zn-MgO plots of MORB and 21 arcs. Small grey dots are single whole rock analyses, whereas large blue and orange dots are median values of the whole rock data for MgO bins ≥0.5 wt.%. Blue dots are used for linear regressions whose equations and R2 values are reported within each plot. Orange dots are excluded from regressions (see text for discussion).

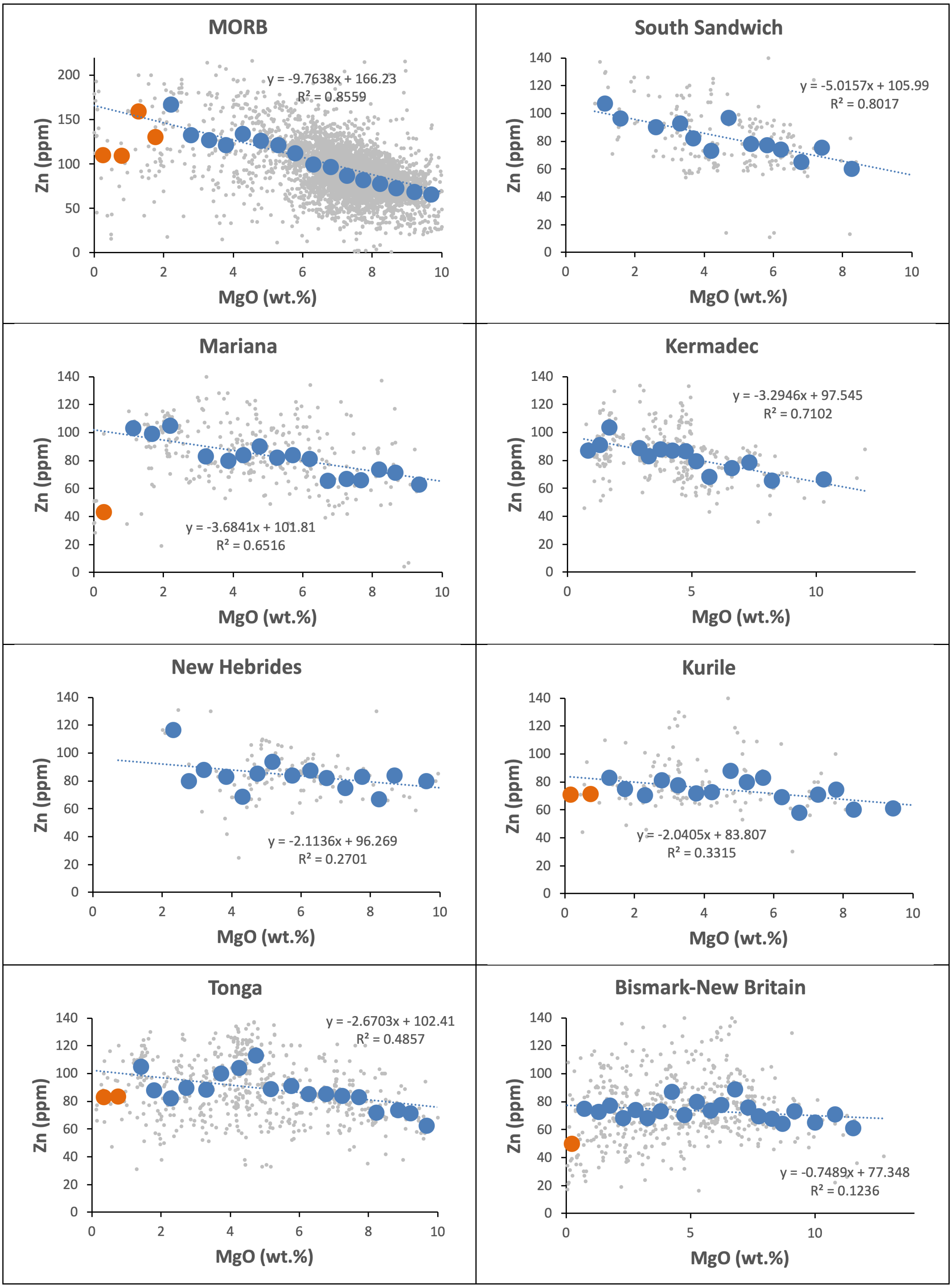

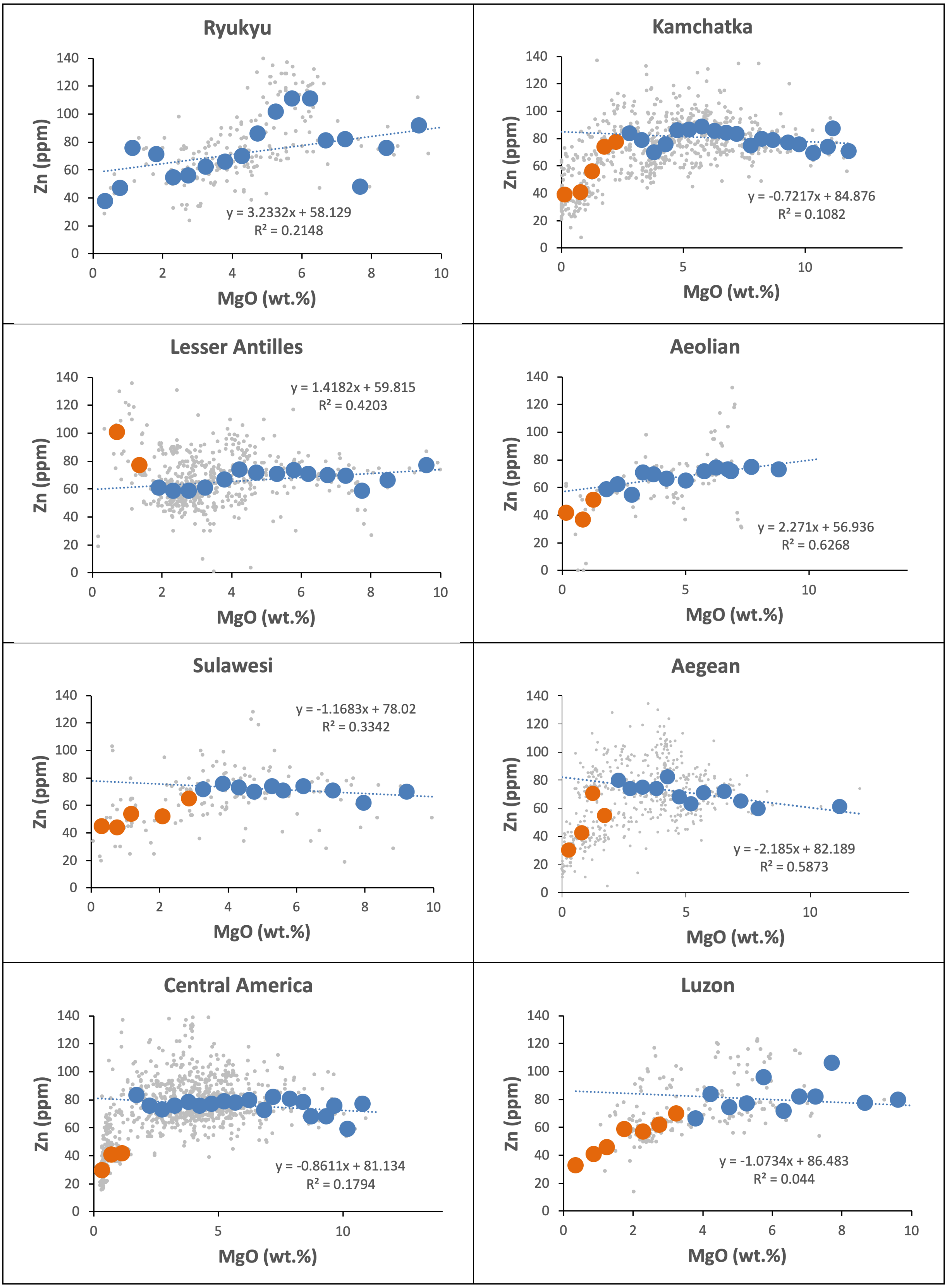
Ryukyu
y = 3.2332x + 58.129
R² = 0.2148
Kamchatka
y = -0.7217x + 84.876
R² = 0.1082
Lesser Antilles
y = 1.4182x + 59.815
R² = 0.4203
Aeolian
y = 2.271x + 56.936
R² = 0.6268
Sulawesi
y = -1.1683x + 78.02
R² = 0.3342
Aegean
y = -2.185x + 82.189
R² = 0.5873
Central America
y = -0.8611x + 81.134
R² = 0.1794
Luzon
y = -1.0734x + 86.483
R² = 0.044
Zn (ppm)
MgO (wt.%)

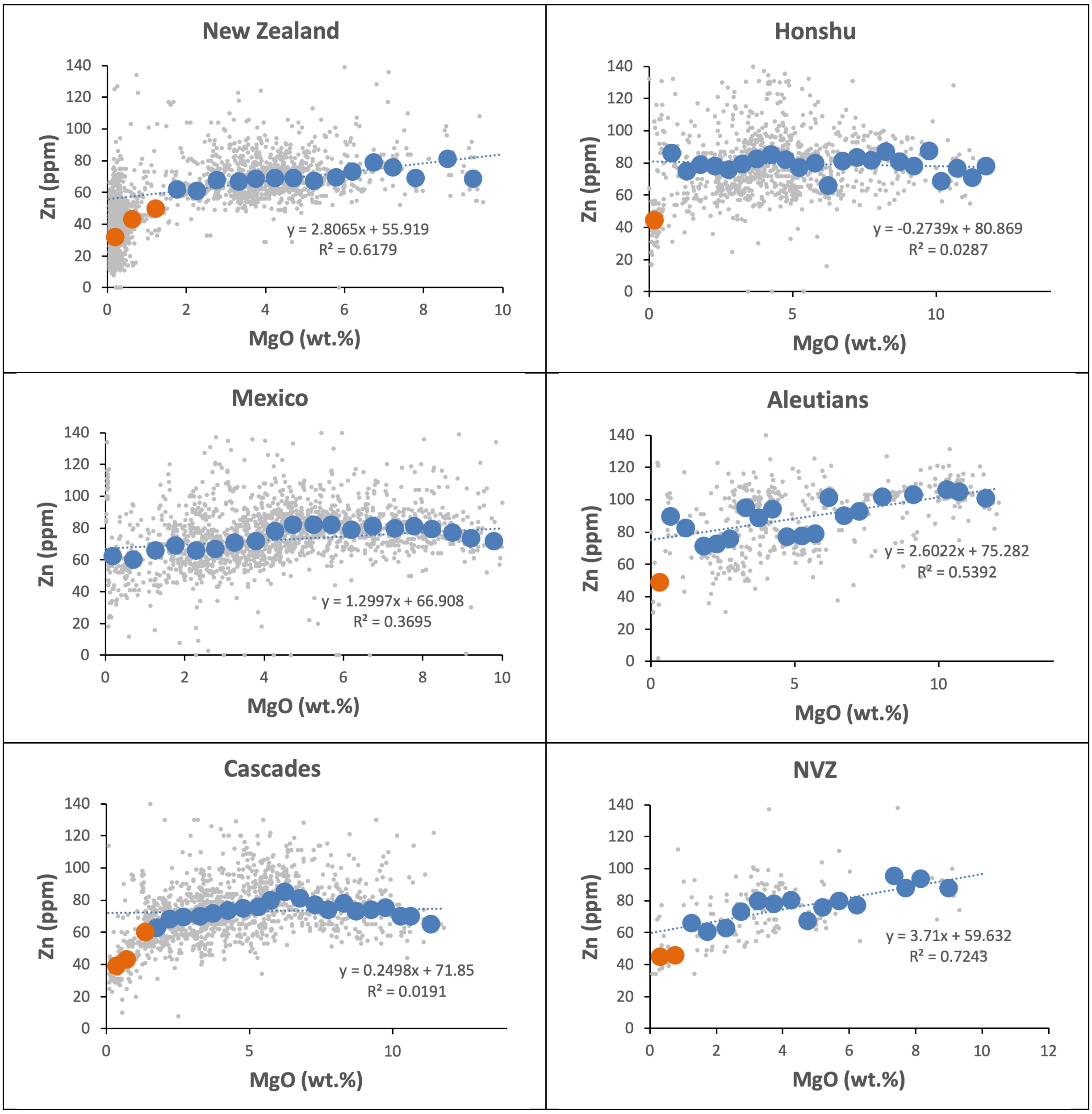

New Zealand
y = 2.8065x + 55.919
R² = 0.6179
Honshu
y = -0.2739x + 80.869
R² = 0.0287
Mexico
y = 1.2997x + 66.908
R² = 0.3695
Aleutians
y = 2.6022x + 75.282
R² = 0.5392
Cascades
y = 0.2498x + 71.85
R² = 0.0191
NVZ
y = 3.71x + 59.632
R² = 0.7243
Zn (ppm)
MgO (wt.%)

Figure S2: MgO-SiO_2 plots of MORB and 21 arcs. Small grey dots are single whole rock analyses, whereas large blue and orange dots are median values of the whole rock data for MgO bins ≥0.5 wt.%. Blue dots are used for linear regressions whose equations and R^2 values are reported within each plot. Orange dots are excluded from regressions (see text for discussion).

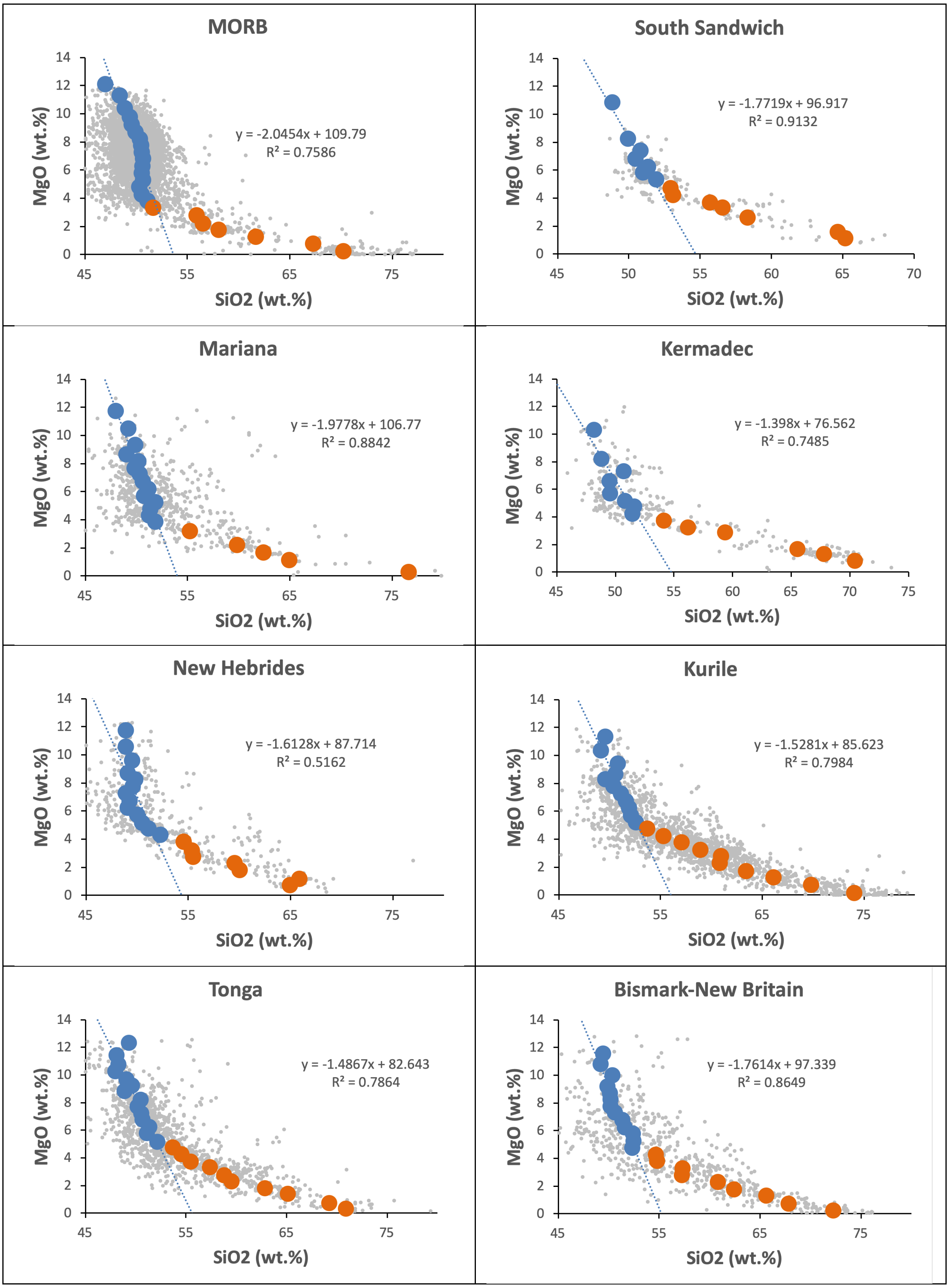

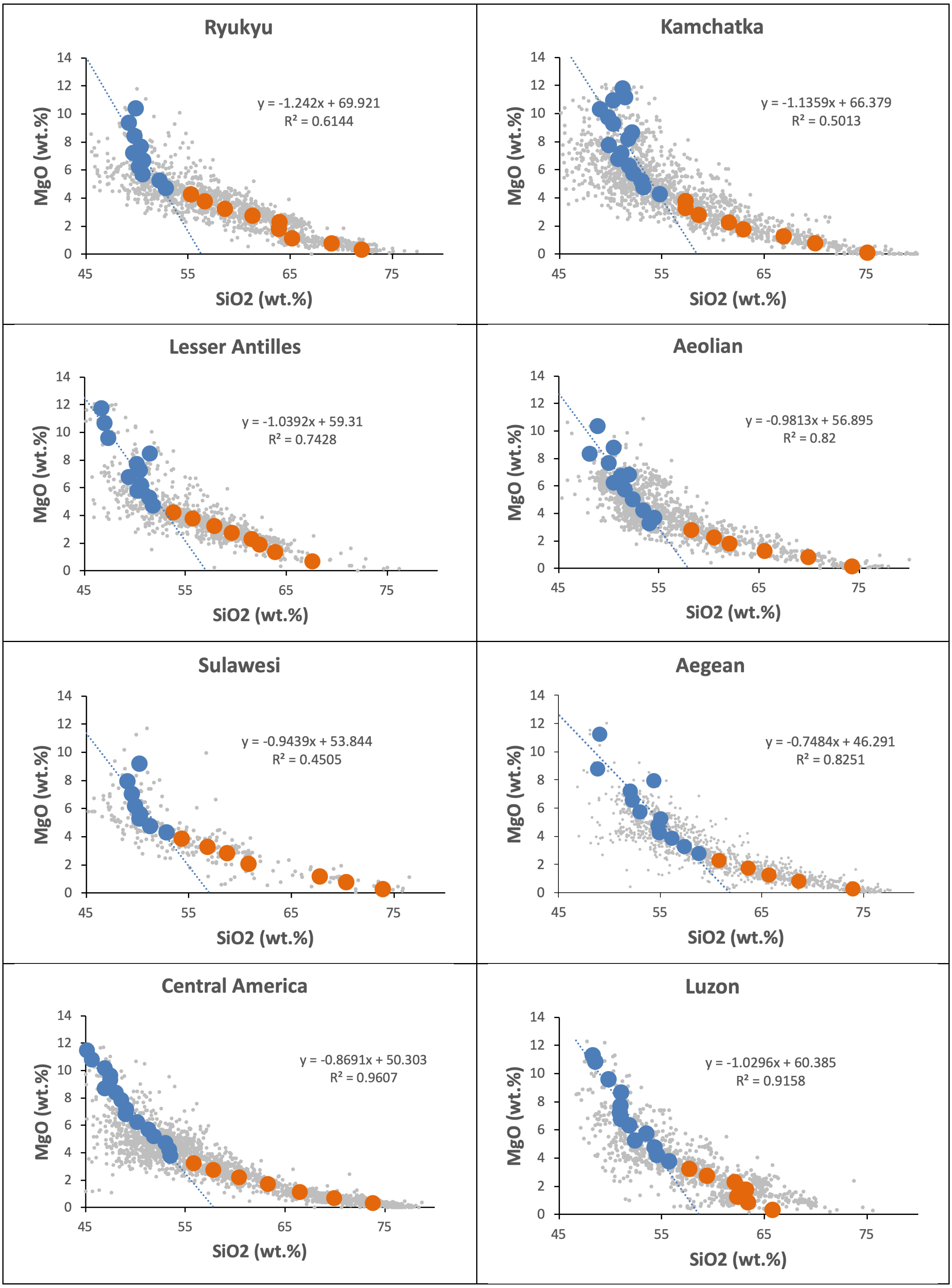

Ryukyu
y = -1.242x + 69.921
R² = 0.6144
Kamchatka
y = -1.1359x + 66.379
R² = 0.5013
Lesser Antilles
y = -1.0392x + 59.31
R² = 0.7428
Aeolian
y = -0.9813x + 56.895
R² = 0.82
Sulawesi
y = -0.9439x + 53.844
R² = 0.4505
Aegean
y = -0.7484x + 46.291
R² = 0.8251
Central America
y = -0.8691x + 50.303
R² = 0.9607
Luzon
y = -1.0296x + 60.385
R² = 0.9158
MgO (wt.%)
SiO2 (wt.%)
0
2
4
6
8
10
12
14
45
55
65
75

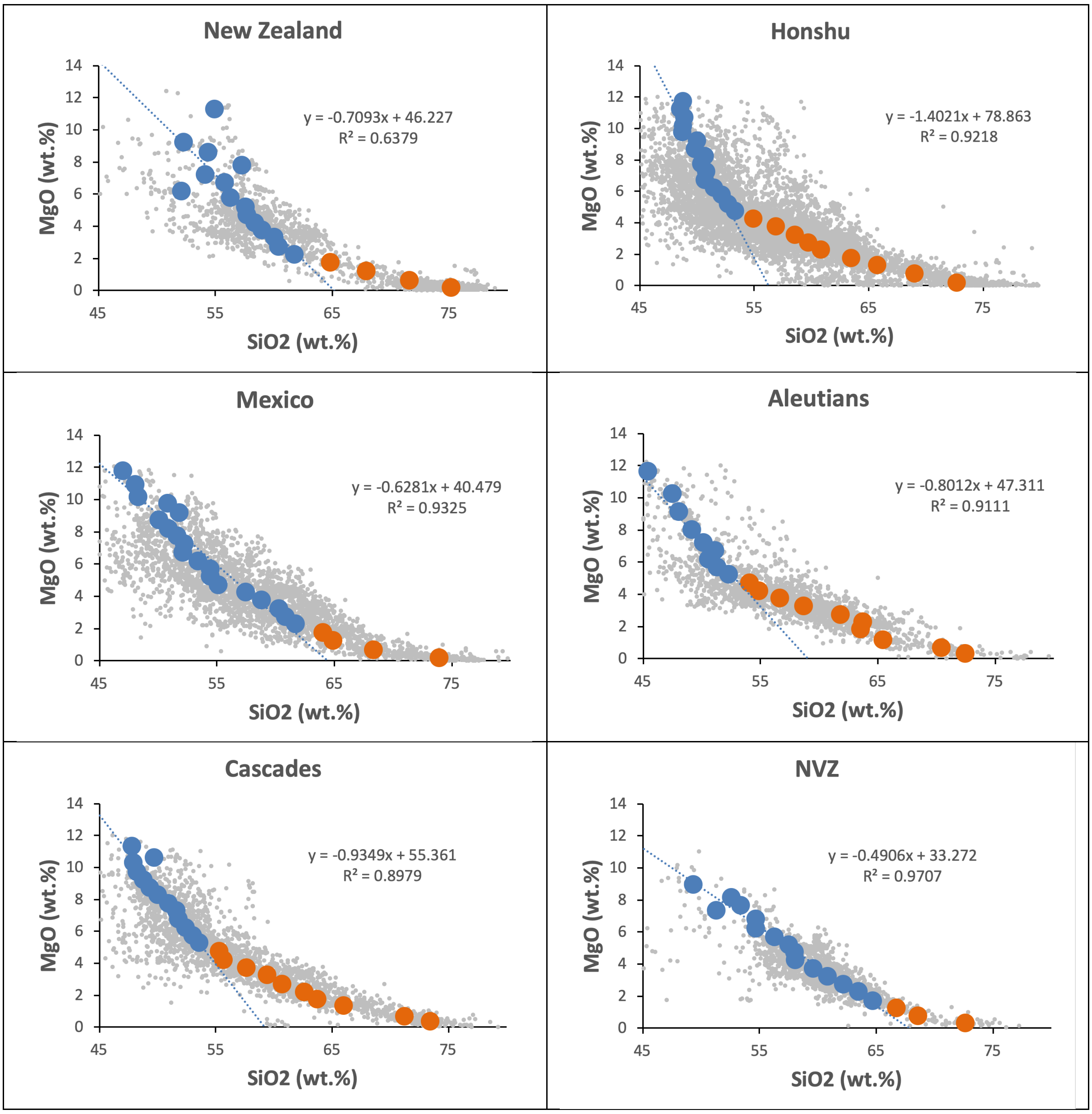
New Zealand
y = -0.7093x + 46.227
R² = 0.6379
Honshu
y = -1.4021x + 78.863
R² = 0.9218
Mexico
y = -0.6281x + 40.479
R² = 0.9325
Aleutians
y = -0.8012x + 47.311
R² = 0.9111
Cascades
y = -0.9349x + 55.361
R² = 0.8979
NVZ
y = -0.4906x + 33.272
R² = 0.9707
MgO (wt.%)
SiO2 (wt.%)
0
2
4
6
8
10
12
14
45
55
65
75

Figure S3: Variations of H_2O solubility (left) and excess H_2O (right) with changing residual melt fraction at 4 different pressures of magma fractionation (see Supplementary Note 2). The starting H_2O content at all pressures is 3.9-4.1 wt.% and the H_2O is considered to behave as completely incompatible. The pressure and melt composition dependency of H_2O solubility in silicate melts are from the parametrization of ref. [1] based on the model of ref. [2]. The different curves are the result of >1000 simulations for ranges of initial H_2O content of 3.9-4.1 wt.% and pressures ranges of 0.19-0.21, 0.39-0.41, 0.59-0.61 and 0.79-0.81 GPa. The kinks of the curves in the left plot indicate the point at which H2O saturation occurs (i.e., when on the right hand plot the curves raise above 0 wt.% excess H_2O). In the right-hand plot negative values indicate H_2O-undersaturated conditions whereas positive ones indicate H_2O-saturated conditions. At 0.2 GPa the magma is already saturated without any crystallization if it has an initial H_2O content between 3.9 and 4.1 wt.%.

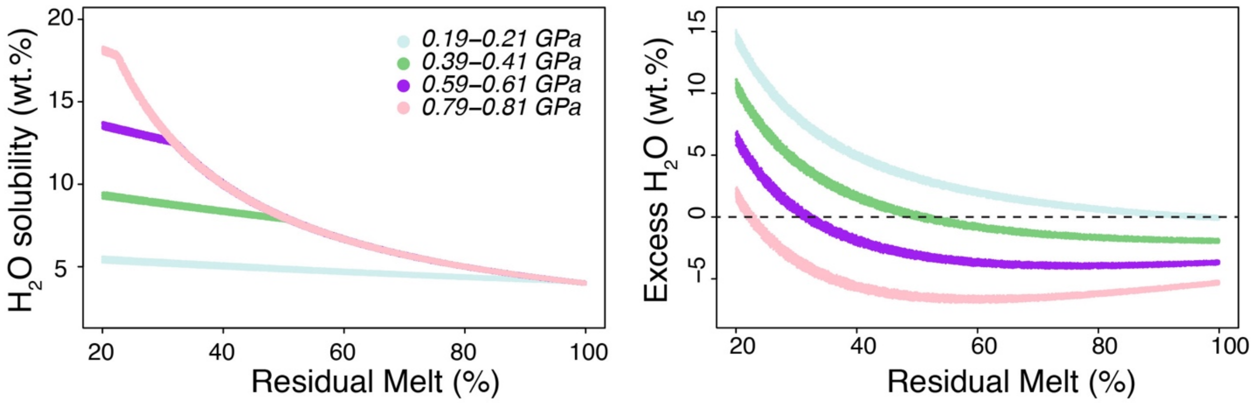

References

[1] Chiaradia, M. & Caricchi, L. Stochastic modelling of deep magmatic controls on porphyry copper deposit endowment. *Scientific Reports* **7**, 44523 (2017).

[2] S. Newman & J. B. Lowenstern. VolatileCalc: a silicate melt–H2O–CO2solution modelwritten in Visual Basic for excel. *Computers & Geosciences* **28**, 597–604 (2002).

Table S1: Partition coefficients for Zn between mineral and silicate melts of different compositions (data and references from GERM and from refs. 5 and 8).

rock	Mineral	K_D	Reference	median	20%	80%
basalt	olivine	0.86	1	0.86	0.774	1.192
		0.8	2			
		1.8	3			
		0.67	4			
		1.04±0.12	5			
	cpx	0.5	1	0.485	0.452	0.494
		0.41	4			
		0.49	3			
		0.48±0.03	5			
	Opx	0.68±0.07	5	0.68		
	plag	0.18	6	0.13	0.118	0.16
		0.11	1			
		0.13	3			
	mt	2.6	7	2.6	2.6	2.6
	gar	0.73-1.026	7a	0.895	0.804	0.994
bas. andesite	olivine	1.2	8	1.2	1.2	1.2
	cpx	0.24	8	0.275	0.254	0.296
		0.31	8			
	amph	0.4	8	0.4	0.4	0.4
	mt	3.1	9	3.1	3.1	3.1
	gar	0.73-1.026	7a	0.895	0.804	0.994
andesite	olivine	1.5	10	2.15	1.264	5.68
		10	11			
		0.91	11			
		2.8	11			
	cpx	6.5	10	4.25	1.8	8.7
		12	10			
		2	12			
		1.5	11			
	opx	3.7	12	3.545	3.074	3.98
		2.6	10			
		4.4	10			
		3.39	11			
	amph	0.42	4	3.3	1.128	6.48
		5	10			
		8.7	10			
		1.6	12			
	plag	0.13	10	0.21	0.154	0.326
		0.25	10			
		0.17	11			
		0.44	12			
	mt	15.5	11	13	8.44	14.5
		5.4	10			
		13	10			
	gar	0.73-1.026	7a	0.895	0.804	0.994
dacite	amph	10.7	11	10.7	10.7	10.7
	biot	16.6	11	18.3	17.28	19.32
		20	4			
	plag	0.26	13	0.51	0.36	0.696
		0.51	13			
		0.82	11			
	mt	26.6	11	26.6	26.6	26.6

References

1 = **Bougault, H. and Hekinian, R. (1974).** Rift valley in the Atlantic Ocean near 36 degrees 50'N; petrology and geochemistry of basalt rocks. *Earth and Planetary Science Letters 24(2): 249-261. doi: 10.1016/0012-821X(74)90103-4.*

2 = **Kloeck, W. and Palme, H. (1988).** Partitioning of siderophile and chalcophile elements between sulfide, olivine, and glass in a naturally reduced basalt from Disko Island, Greenland. *In: Proceedings of the Lunar and Planetary Science Conference, vol.18. Ryder, G. (Editors), Pergamon, New York. 18, 471-483.*

3 = **Paster, T.P., Schauwecker, D.S. and Haskin, L.A. (1974).** The behavior of some trace elements during solidification of the Skaergaard layered series. *Geochimica et Cosmochimica Acta 38(10), 1549-1577. doi: 10.1016/0016-7037(74)90174-4.*

4 = **Matsui, Y., Onuma, N., Nagasawa, H., Higuchi, H. and Banno, S. (1977).** Crystal structure control in trace element partition between crystal and magma. *Tectonics 100: 315-324.*

5 = Le Roux, V., Dasgupta, R., and Lee, C.-T. A. (2011). Mineralogical heterogeneities in the Earth's mantle: Constraints from Mn, Co, Ni and Zn partitioning during partial melting. Earth Planet. Sci. Lett. 307, 395-408.

6 = **Kravuchuk, I.K., Chernysheva, I. and Urosov, S. (1981).** Element distribution between plagioclase and groundmass as an indicator for crystallization conditions of the basalts in the southern vent of Tolbachik. *Geochemistry International 17, 18-24.*

7 = **Lemarchand, F., Benoit, V. and Calais, G. (1987).** Trace element distribution coefficients in alkaline series. *Geochimica et Cosmochimica Acta 51, 1071-1081. doi: 10.1016/0016-7037(87)90201-8.*

8 = **Dostal, J., Dupuy, C., Carron, J.P., Dekerneizon, M.L. and Maury, R.C. (1983).** Partition-Coefficients of Trace-Elements - Application to Volcanic-Rocks of St-Vincent, West-Indies. *Geochimica et Cosmochimica Acta 47(3), 525-533. doi: 10.1016/0016-7037(83)90275-2.*

9 = **Esperança, S., Carlson, R.W., Shirey, S.B. and Smith, D. (1997).** Dating crust-mantle separation: Re-OS isotopic study of mafic xenoliths from central Arizona. *Geology 25, 651-654. doi: 10.1130/0091-7613(1997)¿025<0651:DCMSRO>¿2.3.CO;2.*

9a = Pertermann, M., Hirschmann, M. M., Hametner, K., Günther, D., and Schmidt, M. W. (2004) Experimental determination of trace element partitioning between garnet and silica-rich liquid during anhydrous partial melting of MORB-like eclogite. Geochem. Geophys. Geosys. 5, Q05A01, doi:10.1029/2003GC000638.

10 = **Luhr, J.F. and Carmichael, I.S.E. (1980).** The Colima volcanic complex, Mexico. I: post-caldera andesites from Volcan Colima. *Contributions to Mineralogy and Petrology 71, 343-372.*

11 = **Ewart, A. and Griffin, W.L. (1994).** Application of Proton-Microprobe Data to Trace-Element Partitioning in Volcanic-Rocks. *Chemical Geology 117(1-4), 251-284. doi: 10.1016/0009-2541(94)90131-7.*

12 = **Bacon, C.R. and Druitt, T.H. (1988).** Compositional Evolution of the Zoned Calcalkaline Magma Chamber of Mount-Mazama, Crater Lake, Oregon. *Contributions to Mineralogy and Petrology 98(2), 224-256.*

13 = **Dudas, M.J., Schmitt, R.A. and Harward, M.E. (1971).** Trace element partitioning between volcanic plagioclase and dacitic pyroclastic matrix. *Earth and Planetary Science Letters 11(5), 440-446. doi: 10.1016/0012-821X(71)90206-8.*

Table S2: Input values used for the Monte Carlo modelling detailed in the RStudio codes above. SiO_2-p, MgO-p, Zn-p, SiO_2-t, MgO-t, and Zn-t are the parent (p) and target (t) compositions in wt.% (SiO_2, MgO) and ppm (Zn); plag, cpx, amph, mt, ol, gar are the percent ranges of fractionating minerals (plagioclase, clinopyroxene, amphibole, magnetite, olivine, garnet respectively) used in the model. F is the range of residual melt fraction allowed in the model. For further explanations see Methods section.

Arc	SiO2-p	MgO-p	Zn-p	SiO2-t	MgO-t	Zn-t	plag	cpx	amph	gar	mt	ol	F
S. Sandwich	48.49	11	51	52.4-52.5	3.9-4.1	85-87	38-40	33-35	2-3.5		0.4-0.6	20-23	40-42
Mariana	47.92	12	51	51.9-52.2	3.7-3.9	87-90	36-39	32-34.5	1.5-3		0.4-0.6	22-24	37.5-40
Kermadec	47.25	10.5	63	51.8-52	3.9-4.1	84-85	35-39	32-38	2-4		2-4	19-21	42-45.5
New Hebrid.	46.95	12	71	51.8-52	3.9-4.1	87-88	41-42	24-26	7-10		3-6	20.5-22	28.5-30.5
Kuriles	48.5	11.5	60	53.3-53.5	3.9-4.1	75-76	41-43.5	22-24	6.5-8.5		3-5	23-26	39-43
Tonga	47.5	12	70	52.8-53	3.9-4.1	91-92	39-42	22-25	3-6		3-4.5	26-28	39-43
Bismark-NB	48.45	12	68	52.9-53.1	3.6-3.8	74-75	31-34	26-29	6-9		2-4.5	29-31	47-50
Ryukyu	47.84	10.5	92	52.98-53.18	3.9-4.1	70-72	35-37	24-26	15.5-18.5		4-6	18-19.5	40-42
Kamchatka	48.55-48.95	10.9-11.1	76-79	54.90-54.92	3.9-4.1	80-84	25-30.5	15-41	10-15	0-0.1	4-6	25-31	50-56
Lesser Antill.	45.67-45.87	11.65-11.85	74-79	53.12-53.23	3.9-4.1	65-68	24-28	15-21	16-20	0-0.6	4-8	25-29	37-50
Aeolian	47.08-47.48	10.4-10.6	80-83	54.82-55.02	2.9-3.1	63-64	32-38	12-18	18-22	0-0.5	3-6	20-28	39-45
Sulawesi	47.41-47.61	8.9-9.1	66-69	52.61-52.95	3.9-4.1	73-74	29-42	15-30	10-20	0-1	3-7	15-25	51-57
Luzon	47.38-47.58	11.4-11.6	73-77	53.96-54.16	3.6-3.8	82-84	22-28	21-27.5	10-20	1-2.5	2-6	24-28	45-50
Central Am.	45.12-45.32	10.9-11.1	71-73	53.41-53.61	3.7-3.9	76-80	32-38	8-16	12-18	1-5	3.5-8.5	21-25	37-43
Aegean	46.29-46.69	11.4-11.6	55-59	58.0-58.3	2.65-2.85	75-78	25-41	10-14	11-15	3.5-5	5-7	22-27	32-35
New Zealand	49.66	11	87	62.25-62.45	1.9-2.1	61-63	30-34	8-12	12-16	2-6	5-12	28-32	43-47
Honshu	47.69	12	78	53.29-53.49	3.9-4.1	79-82	16-20	29-32	16-20	3-7	2-6	20-24	43-48
Mexico	45.14-45-54	11.9-12.1	81-84	61.16-61.36	1.9-2.1	69-71	9.5-12.5	28-32	22-26	8-11	7.5-11	12-15	24-30
Aleutians	44.6-44.8	11.4-11.6	104-106	53.33-53.53	4.4-4.6	86-88	24-28	10-14	18-22	7-11	7-9	20-24	40-42
Cascades	46.92	11.5	75	53.77-53.97	4.9-5.1	72-74	13-17	20-24	22-26	8.5-11	5-7.5	18-22	45-50
NVZ	49.37-49.57	8.9-9.1	92-95	63.64-63.84	1.9-2.1	66-67	14-18	10-16	30-38	9-13	8-11	12-16	41-45
MORB	48.65	10.7	58	51.86-52.06	2.9-3.1	134-136	53-57	25-29	0-1	0	0-1	18-20.5	27-32

Table S3: Ranges of Zn K_D values for mineral-melt used for Monte Carlo modelling (RStudio codes above) taking into account the variable values of Zn K_D for basaltic to andesitic melts (Table S2). Also reported are the compositional ranges used in the model for SiO_2 and MgO for the fractionating minerals. These ranges are from the mineral compositions from experiments of Müntener et al. (2001) and Alonso-Perez et al. (2009). MgO values are correlated with the SiO_2 ranges through the best fit equations reported. The compositional ranges for Zn were determined from the ranges of KD values and the Zn contents of the parental melt (Table S5).

Mineral	K_D	SiO2 (wt.%)	MgO (wt.%)	Zn (ppm)
Olivine	0.83-1.2	40.1-40.3	10.5*SiO_2_ol - 374.95	Zn_{par}*KD_{ol}
Clinopyroxene	0.45-0.55	48-54	0.9523*SiO_2_cpx-32.253	Zn_{par}*KD_{cpx}
Amphibole	0.4-3.3	40.7-43.5	7.3-15	Zn_{par}*KD_{amph}
Spinel	7-9.1	0.004-0.22	4-17	Zn_{par}*KD_{mt}
Plagioclase	0.13-0.21	43.5-49	0.066	Zn_{par}*KD_{plag}
Garnet	0.73-1.03	37.4-41.2	3.1738*SiO_2_gar-115.05	Zn_{par}*KD_{gar}

References

Müntener, O., Kelemen, P. B. & Grove, T. L. The role of H2O during crystallization of primitive arc magmas under uppermost mantle conditions and genesis of igneous pyroxenites: an experimental study. *Contrib Mineral Petrol* **141**, 643–658 (2001).
Alonso-Perez, R., Müntener, O. & Ulmer, P. Igneous garnet and amphibole fractionation in the roots of island arcs: experimental constraints on andesitic liquids. *Contributions to Mineralogy and Petrology* **157**, 541–558 (2009).

Table S4: Fractionating mineral assemblages calculated from the averages of the mineral fractions (and associated standard deviation, SD) obtained with the Monte Carlo simulations of the MgO-SiO2 and Zn-MgO trends (Supplementary Dataset). Abbreviations: Gar = garnet; Amph = amphibole; Plag = plagioclase; Oliv = olivine; Cpx = clinopyroxene; Mt = magnetite; F = fraction of remaining melt after mineral fractionation.

Arc	Crust thickness (km)	error	Gar	SD	Amph	SD	Plag	SD	Oliv	SD	Cpx	SD	Mt	SD	F	SD
S. Sandwich	11.8	0.1			0.0274	0.0001	0.3916	0.0002	0.2130	0.0001	0.3408	0.0002	0.0050	0.0000	0.4109	0.0002
Mariana	14.5	1			0.0227	0.0000	0.3762	0.0001	0.2313	0.0001	0.3341	0.0001	0.0050	0.0000	0.3869	0.0001
Kermadec	15	3			0.0302	0.0001	0.3684	0.0003	0.2002	0.0002	0.3466	0.0003	0.0321	0.0001	0.4385	0.0002
New Hebrid.	15.6	0.2			0.0882	0.0006	0.4155	0.0003	0.2121	0.0003	0.2510	0.0009	0.0450	0.0010	0.2967	0.0003
Kuriles	18.3	0.9			0.0756	0.0001	0.4238	0.0001	0.2470	0.0002	0.2311	0.0003	0.0395	0.0002	0.4134	0.0003
Tonga	20	0			0.0458	0.0001	0.4054	0.0001	0.2707	0.0001	0.2359	0.0002	0.0374	0.0002	0.4118	0.0001
Bismark-NB	22.5	6.5			0.0756	0.0001	0.3244	0.0002	0.3003	0.0001	0.2745	0.0001	0.0349	0.0001	0.4857	0.0002
Ryukyu	24.5	3.4			0.1728	0.0004	0.3616	0.0003	0.1871	0.0002	0.2514	0.0006	0.0500	0.0003	0.4114	0.0004
Kamchatka	24.6	5.4	0.00051	0.00005	0.1239	0.0018	0.2785	0.0016	0.2798	0.0011	0.2438	0.0030	0.0499	0.0008	0.5332	0.0013
Lesser Antilles	24.7	0.7	0.00310	0.00017	0.1809	0.0012	0.2620	0.0014	0.2697	0.0010	0.1808	0.0020	0.0662	0.0007	0.4533	0.0016
Aeolian	24.9	1	0.00251	0.00006	0.2001	0.0005	0.3506	0.0007	0.2310	0.0009	0.1503	0.0003	0.0456	0.0005	0.4244	0.0008
Sulawesi	27.4	2.2	0.00506	0.00010	0.1461	0.0011	0.3555	0.0015	0.1966	0.0010	0.2145	0.0023	0.0521	0.0003	0.5402	0.0008
Luzon	27.8	4.5	0.01747	0.00027	0.1516	0.0005	0.2526	0.0011	0.2617	0.0007	0.2440	0.0012	0.0420	0.0006	0.4740	0.0008
Centr. America	28	7	0.03233	0.00014	0.1535	0.0007	0.3544	0.0007	0.2330	0.0002	0.1270	0.0010	0.0654	0.0010	0.3980	0.0006
Aegean	28.2	0.6	0.04253	0.00013	0.1300	0.0011	0.3785	0.0017	0.2435	0.0008	0.1205	0.0013	0.0595	0.0007	0.3360	0.0005
New Zealand	28.6	3.2	0.03960	0.00060	0.1397	0.0002	0.3197	0.0005	0.2980	0.0002	0.0993	0.0004	0.0860	0.0009	0.4517	0.0006
Honshu	29.2	2.6	0.05166	0.00075	0.1817	0.0010	0.1819	0.0004	0.2240	0.0007	0.3061	0.0003	0.0403	0.0002	0.4567	0.0007
Mexico	30.3	5.5	0.09483	0.00090	0.2399	0.0006	0.1100	0.0011	0.1359	0.0010	0.2990	0.0009	0.0924	0.0008	0.2703	0.0011
Aleutians	37.5	2.5	0.08932	0.00075	0.1987	0.0009	0.2589	0.0008	0.2215	0.0009	0.1190	0.0007	0.0796	0.0004	0.4099	0.0005
Cascades	38.8	1.9	0.09834	0.00034	0.2411	0.0002	0.1518	0.0004	0.2018	0.0006	0.2217	0.0006	0.0616	0.0002	0.4793	0.0008
NVZ	42	9.8	0.11037	0.00163	0.3413	0.0037	0.1610	0.0011	0.1398	0.0010	0.1317	0.0019	0.0939	0.0006	0.4336	0.0006
MORB	6.5	0.5			0.0049	0.0002	0.5489	0.0007	0.1932	0.0003	0.2704	0.0009	0.0046	0.0001	0.2892	0.0003

Table S5: Calculations to determine the amounts of H2O stored in crystallized cumulate of arcs (Column E) for the remaining melt fractions reported in Column C. This is based on the assumption that all H2O is stored in amphibole and that amphibole has a H2O content of 2 wt.%. Amphibole fraction (Column B) is from Table S5. Column F represents the % of the H2O stored in cumulate with respect to the initial H2O content of the primary basalt (assumed to be 4 wt.%: Plank et al., 2013). Abbreviations: Amph = amphibole; fract = fraction; cum = cumulate.

A	B	C	D	E	F
**Arc**	**Amphibole fraction**	**F melt**	**Amph in cumulate**	**H2O in cumulate**	**% of initial H2O lost to cumulate**
S. Sandwich	0.0274	0.4109	0.016	0.032	0.81
Mariana	0.0227	0.3869	0.014	0.028	0.69
Kermadec	0.0302	0.4385	0.017	0.034	0.85
New Hebrides	0.0882	0.2967	0.062	0.124	3.10
Kuriles	0.0756	0.4134	0.044	0.089	2.22
Tonga	0.0458	0.4118	0.027	0.054	1.35
Bismark-New Britain	0.0756	0.4857	0.039	0.078	1.94
Ryukyu	0.1728	0.4114	0.102	0.203	5.09
Kamchatka	0.1239	0.5332	0.058	0.116	2.89
Lesser Antilles	0.1809	0.4533	0.099	0.198	4.94
Aeolian	0.2001	0.4244	0.115	0.230	5.76
Sulawesi	0.1461	0.5402	0.067	0.134	3.36
Luzon	0.1516	0.4740	0.080	0.159	3.99
Central America	0.1535	0.3980	0.092	0.185	4.62
Aegean	0.1300	0.3360	0.086	0.173	4.32
New Zealand	0.1397	0.4517	0.077	0.153	3.83
Honshu	0.1817	0.4567	0.099	0.197	4.94
Mexico	0.2399	0.2703	0.175	0.350	8.75
Aleutians	0.1987	0.4099	0.117	0.235	5.85
Cascades	0.2411	0.4793	0.126	0.251	6.28
Ecuador	0.3413	0.4336	0.193	0.387	9.67
MORB	0.0049	0.2892	0.004	0.007	0.18

Equations used to obtain columns D, E, F

Column D: Amph_cum=((1-F_melt)*(Amph_fract*100)/100 (in %)
Column E: H2O_cum =2*Amph_cum. (in wt.%)
Column F: % of initial H2O lost to cumulate =H2O_cum/4*100 (in %)

Supplementary Note 1: RStudio Code for Monte Carlo simulations of the Zn-MgO and MgO-SiO_2 correlations

```
ns= 5000000
################INPUT PARAMETERS ###############
#Parent composition SOUTH SANDWICH
SiO2_par<- runif(min=48.49, max=48.49, ns) #ppm
MgO_par<- runif(min=11, max=11, ns) #ppm
ZnO_par<- runif(min=0.00632, max=0.00632, ns) #ppm


#Target composition SOUTH SANDWICH
SiO2_target_min<- 52.4
SiO2_target_max<- 52.5
MgO_target_min<- 3.9
MgO_target_max<- 4.1
ZnO_target_min<- 0.0106
ZnO_target_max<- 0.0108

Cmelt_SiO2_target1<- runif(min=SiO2_target_min, max=SiO2_target_max, ns)
Cmelt_MgO_target1<- runif(min = MgO_target_min, max = MgO_target_max, ns)
Cmelt_ZnO_target1<- runif(min = ZnO_target_min, max = ZnO_target_max, ns)



#####  MINERAL COMPOSITIONS   ######

#### KDs MINERALS
kd_ol<- runif(min = 0.83, max = 1.2, ns)
kd_cpx<- runif(min = 0.45, max = 0.55, ns)
kd_amph<- runif(min = 0.4, max = 3.3, ns)
kd_mt<- runif(min = 3, max = 9.1, ns)
kd_plag<- runif(min = 0.13, max = 0.21, ns)
kd_gar<- runif(min=0.73, max = 1.03, ns)
kd_opx<- runif(min=2.34, max = 4.14, ns)



# Olivine composition
SiO2_ol<- runif(min = 40.1, max = 40.3, ns)
MgO_ol<- 10.5*SiO2_ol -374.95
ZnO_ol<- ZnO_par*kd_ol

# Opx composition
SiO2_opx<- runif(min = 50.1, max = 56, ns)
MgO_opx<- 1.0815*SiO2_opx - 28.771
ZnO_opx<- ZnO_par*kd_opx


# Cpx composition
SiO2_cpx<- runif(min = 48, max = 54, ns)
MgO_cpx<- 0.9523*SiO2_cpx-32.253
ZnO_cpx<- ZnO_par*kd_cpx


# Amph composition
SiO2_amph<- runif(min = 40.7, max = 43.5, ns)
MgO_amph<- runif(min = 7.3, max = 15, ns)
ZnO_amph<- ZnO_par*kd_amph
```

```
# Plag composition
SiO2_plag<- runif(min = 43.5, max = 49, ns)
MgO_plag<- runif(min = 0.066, max = 0.066, ns)
ZnO_plag<- ZnO_par*kd_plag


# Magnetite composition
SiO2_mt<- runif(min = 0.04, max = 0.22, ns)
MgO_mt<- runif(min = 4, max = 17, ns)
ZnO_mt<- ZnO_par*kd_mt


# Garnet composition
SiO2_gar<- runif(min = 37.4, max = 41.2, ns)
MgO_gar<- 3.1738*SiO2_gar-115.05
ZnO_gar<- ZnO_par*kd_gar


#Fractionating assemblage
plag<- runif(min=0.38, max=0.4, ns)
cpx<- runif(min=0.33, max=0.35, ns)
amph<- runif(min=0.02, max=0.035, ns)
mt<- runif(min=0.004, max = 0.006, ns)
ol<- runif(min=0.2, max = 0.23, ns)
gar<- runif(min=0, max = 0, ns)
opx<- runif(min=0, max = 0, ns)


#Mass fractionating

#Oxides wt% in fractionating assemblage
SiO2_fract<- plag*SiO2_plag+cpx*SiO2_cpx+amph*SiO2_amph+mt*SiO2_mt+ol*SiO2_ol
ZnO_fract<- plag*ZnO_plag+cpx*ZnO_cpx+amph*ZnO_amph+mt*ZnO_mt+ol*ZnO_ol
MgO_fract<- plag*MgO_plag+cpx*MgO_cpx+amph*MgO_amph+mt*MgO_mt+ol*MgO_ol


#Mass liquid
Mass_liquid<- runif(min = 40, max = 42, ns)


#Mass solid
Mass_solid<- 100-Mass_liquid

#F parameter=fraction liquid remaining
Fraction_Liquid<- Mass_liquid/100

#Oxides wt.% in residual melt not normalized
SiO2_resmelt<- SiO2_par-SiO2_fract*Mass_solid/100
MgO_resmelt<- MgO_par-MgO_fract*Mass_solid/100
ZnO_resmelt<-ZnO_par-ZnO_fract*Mass_solid/100


#Oxides wt.% in residual melt normalized
SiO2_melt<- SiO2_resmelt/Mass_liquid*100
MgO_melt<- MgO_resmelt/Mass_liquid*100
ZnO_melt<- ZnO_resmelt/Mass_liquid*100
```

```
#############################################################LOGICAL TESTS
#sum of fractionating minerals must be between 0.95 and 1.05

#Parent composition
SiO2_par1<- SiO2_par[(opx+gar+plag+cpx+amph+mt+ol)>0.95 & (opx+gar+plag+cpx+amph+mt+ol)<1.05]
MgO_par1<- MgO_par[(opx+gar+plag+cpx+amph+mt+ol)>0.95 & (opx+gar+plag+cpx+amph+mt+ol)<1.05]
ZnO_par1<- ZnO_par[(opx+gar+plag+cpx+amph+mt+ol)>0.95 & (opx+gar+plag+cpx+amph+mt+ol)<1.05]


#Target composition
Cmelt_SiO2_target1_1<- Cmelt_SiO2_target1[(opx+gar+plag+cpx+amph+mt+ol)>0.95 &
(opx+gar+plag+cpx+amph+mt+ol)<1.05]
Cmelt_MgO_target1_1<- Cmelt_MgO_target1[(opx+gar+plag+cpx+amph+mt+ol)>0.95 &
(opx+gar+plag+cpx+amph+mt+ol)<1.05]
Cmelt_ZnO_target1_1<- Cmelt_ZnO_target1[(opx+gar+plag+cpx+amph+mt+ol)>0.95 &
(opx+gar+plag+cpx+amph+mt+ol)<1.05]


# Olivine composition
SiO2_ol1<- SiO2_ol[(opx+gar+plag+cpx+amph+mt+ol)>0.95 & (opx+gar+plag+cpx+amph+mt+ol)<1.05]
MgO_ol1<- MgO_ol[(opx+gar+plag+cpx+amph+mt+ol)>0.95 & (opx+gar+plag+cpx+amph+mt+ol)<1.05]
ZnO_ol1<- ZnO_ol[(opx+gar+plag+cpx+amph+mt+ol)>0.95 & (opx+gar+plag+cpx+amph+mt+ol)<1.05]


# Cpx composition
SiO2_cpx1<- SiO2_cpx[(opx+gar+plag+cpx+amph+mt+ol)>0.95 & (opx+gar+plag+cpx+amph+mt+ol)<1.05]
MgO_cpx1<- MgO_cpx[(opx+gar+plag+cpx+amph+mt+ol)>0.95 & (opx+gar+plag+cpx+amph+mt+ol)<1.05]
ZnO_cpx1<- ZnO_cpx[(opx+gar+plag+cpx+amph+mt+ol)>0.95 & (opx+gar+plag+cpx+amph+mt+ol)<1.05]


# Amph composition
SiO2_amph1<- SiO2_amph[(opx+gar+plag+cpx+amph+mt+ol)>0.95 &
(opx+gar+plag+cpx+amph+mt+ol)<1.05]
MgO_amph1<- MgO_amph[(opx+gar+plag+cpx+amph+mt+ol)>0.95 &
(opx+gar+plag+cpx+amph+mt+ol)<1.05]
ZnO_amph1<- ZnO_amph[(opx+gar+plag+cpx+amph+mt+ol)>0.95 &
(opx+gar+plag+cpx+amph+mt+ol)<1.05]


# Plag composition
SiO2_plag1<- SiO2_plag[(opx+gar+plag+cpx+amph+mt+ol)>0.95 & (opx+gar+plag+cpx+amph+mt+ol)<1.05]
MgO_plag1<- MgO_plag[(opx+gar+plag+cpx+amph+mt+ol)>0.95 & (opx+gar+plag+cpx+amph+mt+ol)<1.05]
ZnO_plag1<- ZnO_plag[(opx+gar+plag+cpx+amph+mt+ol)>0.95 & (opx+gar+plag+cpx+amph+mt+ol)<1.05]


# Magnetite composition
SiO2_mt1<- SiO2_mt[(opx+gar+plag+cpx+amph+mt+ol)>0.95 & (opx+gar+plag+cpx+amph+mt+ol)<1.05]
MgO_mt1<- MgO_mt[(opx+gar+plag+cpx+amph+mt+ol)>0.95 & (opx+gar+plag+cpx+amph+mt+ol)<1.05]
ZnO_mt1<- ZnO_mt[(opx+gar+plag+cpx+amph+mt+ol)>0.95 & (opx+gar+plag+cpx+amph+mt+ol)<1.05]

# Garnet composition
SiO2_gar1<- SiO2_gar[(opx+gar+plag+cpx+amph+mt+ol)>0.95 & (opx+gar+plag+cpx+amph+mt+ol)<1.05]
MgO_gar1<- MgO_gar[(opx+gar+plag+cpx+amph+mt+ol)>0.95 & (opx+gar+plag+cpx+amph+mt+ol)<1.05]
ZnO_gar1<- ZnO_gar[(opx+gar+plag+cpx+amph+mt+ol)>0.95 & (opx+gar+plag+cpx+amph+mt+ol)<1.05]

# Opx composition
SiO2_opx1<- SiO2_opx[(opx+gar+plag+cpx+amph+mt+ol)>0.95 & (opx+gar+plag+cpx+amph+mt+ol)<1.05]
```

```
MgO_opx1<- MgO_opx[(opx+gar+plag+cpx+amph+mt+ol)>0.95 & (opx+gar+plag+cpx+amph+mt+ol)<1.05]
ZnO_opx1<- ZnO_opx[(opx+gar+plag+cpx+amph+mt+ol)>0.95 & (opx+gar+plag+cpx+amph+mt+ol)<1.05]

#Fractionating assemblage
plag1<- plag[(opx+gar+plag+cpx+amph+mt+ol)>0.95 & (opx+gar+plag+cpx+amph+mt+ol)<1.05]
cpx1<- cpx[(opx+gar+plag+cpx+amph+mt+ol)>0.95 & (opx+gar+plag+cpx+amph+mt+ol)<1.05]
amph1<- amph[(opx+gar+plag+cpx+amph+mt+ol)>0.95 & (opx+gar+plag+cpx+amph+mt+ol)<1.05]
mt1<- mt[(opx+gar+plag+cpx+amph+mt+ol)>0.95 & (opx+gar+plag+cpx+amph+mt+ol)<1.05]
ol1<- ol[(opx+gar+plag+cpx+amph+mt+ol)>0.95 & (opx+gar+plag+cpx+amph+mt+ol)<1.05]
gar1<- gar[(opx+gar+plag+cpx+amph+mt+ol)>0.95 & (opx+gar+plag+cpx+amph+mt+ol)<1.05]
opx1<- opx[(opx+gar+plag+cpx+amph+mt+ol)>0.95 & (opx+gar+plag+cpx+amph+mt+ol)<1.05]

#Mass fractionating

#Oxides wt% in fractionating assemblage
SiO2_fract1<- SiO2_fract[(opx+gar+plag+cpx+amph+mt+ol)>0.95 & (opx+gar+plag+cpx+amph+mt+ol)<1.05]
ZnO_fract1<- ZnO_fract[(opx+gar+plag+cpx+amph+mt+ol)>0.95 & (opx+gar+plag+cpx+amph+mt+ol)<1.05]
MgO_fract1<- MgO_fract[(opx+gar+plag+cpx+amph+mt+ol)>0.95 & (opx+gar+plag+cpx+amph+mt+ol)<1.05]


#Mass liquid
Mass_liquid1<- Mass_liquid[(opx+gar+plag+cpx+amph+mt+ol)>0.95 &
(opx+gar+plag+cpx+amph+mt+ol)<1.05]

#Mass solid
Mass_solid1<- Mass_solid[(opx+gar+plag+cpx+amph+mt+ol)>0.95 &
(opx+gar+plag+cpx+amph+mt+ol)<1.05]

#F parameter=fraction liquid remaining
Fraction_Liquid1<- Fraction_Liquid[(opx+gar+plag+cpx+amph+mt+ol)>0.95 &
(opx+gar+plag+cpx+amph+mt+ol)<1.05]

#Oxides wt.% in residual melt not normalized
SiO2_resmelt1<- SiO2_resmelt[(opx+gar+plag+cpx+amph+mt+ol)>0.95 &
(opx+gar+plag+cpx+amph+mt+ol)<1.05]
MgO_resmelt1<- MgO_resmelt[(opx+gar+plag+cpx+amph+mt+ol)>0.95 &
(opx+gar+plag+cpx+amph+mt+ol)<1.05]
ZnO_resmelt1<- ZnO_resmelt[(opx+gar+plag+cpx+amph+mt+ol)>0.95 &
(opx+gar+plag+cpx+amph+mt+ol)<1.05]


#Oxides wt.% in residual melt normalized
SiO2_melt1<- SiO2_melt[(opx+gar+plag+cpx+amph+mt+ol)>0.95 & (opx+gar+plag+cpx+amph+mt+ol)<1.05]
MgO_melt1<- MgO_melt[(opx+gar+plag+cpx+amph+mt+ol)>0.95 & (opx+gar+plag+cpx+amph+mt+ol)<1.05]
ZnO_melt1<- ZnO_melt[(opx+gar+plag+cpx+amph+mt+ol)>0.95 & (opx+gar+plag+cpx+amph+mt+ol)<1.05]


########################CONDITIONS#################

SiO2_high<- SiO2_target_max
SiO2_low<- SiO2_target_min
MgO_high<- MgO_target_max
MgO_low<- MgO_target_min
ZnO_high<- ZnO_target_max
ZnO_low<- ZnO_target_min

amph_target<-amph1[MgO_melt1>MgO_low & MgO_melt1<MgO_high & SiO2_melt1>SiO2_low &
SiO2_melt1<SiO2_high& ZnO_melt1>ZnO_low & ZnO_melt1<ZnO_high]
```

```
Fraction_liquid_target<- Fraction_Liquid1[MgO_melt1>MgO_low & MgO_melt1<MgO_high &
SiO2_melt1>SiO2_low & SiO2_melt1<SiO2_high& ZnO_melt1>ZnO_low & ZnO_melt1<ZnO_high]
plag_target<- plag1[MgO_melt1>MgO_low & MgO_melt1<MgO_high & SiO2_melt1>SiO2_low &
SiO2_melt1<SiO2_high& ZnO_melt1>ZnO_low & ZnO_melt1<ZnO_high]
cpx_target<- cpx1[MgO_melt1>MgO_low & MgO_melt1<MgO_high & SiO2_melt1>SiO2_low &
SiO2_melt1<SiO2_high& ZnO_melt1>ZnO_low & ZnO_melt1<ZnO_high]
ol_target<- ol1[MgO_melt1>MgO_low & MgO_melt1<MgO_high & SiO2_melt1>SiO2_low &
SiO2_melt1<SiO2_high& ZnO_melt1>ZnO_low & ZnO_melt1<ZnO_high]
mt_target<- mt1[MgO_melt1>MgO_low & MgO_melt1<MgO_high & SiO2_melt1>SiO2_low &
SiO2_melt1<SiO2_high& ZnO_melt1>ZnO_low & ZnO_melt1<ZnO_high]
gar_target<- gar1[MgO_melt1>MgO_low & MgO_melt1<MgO_high & SiO2_melt1>SiO2_low &
SiO2_melt1<SiO2_high& ZnO_melt1>ZnO_low & ZnO_melt1<ZnO_high]
opx_target<- opx1[MgO_melt1>MgO_low & MgO_melt1<MgO_high & SiO2_melt1>SiO2_low &
SiO2_melt1<SiO2_high& ZnO_melt1>ZnO_low & ZnO_melt1<ZnO_high]


hist(opx_target)
hist(gar_target)
hist(amph_target)
hist(plag_target)
hist(cpx_target)
hist(ol_target)
hist(mt_target)
hist(Fraction_liquid_target)

mean(opx_target)
mean(gar_target)
mean(amph_target)
mean(plag_target)
mean(ol_target)
mean(cpx_target)
mean(mt_target)
mean(Fraction_liquid_target)
```

Supplementary Note 2: RStudio Code for Monte Carlo simulations of H2O solubility in fractionating magmas with initial H_2O contents variable between 3.9 and 4.1 wt.%.

```
ns= 500000
#########INPUT PARAMETERS

H2O_p<- runif(min = 3.9, max = 4.1, ns)
P<- runif(min = 1.9, max = 8.1, ns)
ResMel<- runif(min = 20, max = 100, ns)
H2Ores<-100*H2O_p*ResMel^-1
rH2O<- 0.00000163*P^2+0.00002024*P-0.00000718
sH2O<- -0.00088884*P^2-0.0110706*P+0.00391827
tH2O<- 0.05908101*P^2+1.91102201*P+1.79358317
H2Osolub<- rH2O*ResMel^2+sH2O*ResMel+tH2O
logicH2Ores<- ifelse(H2Ores>H2Osolub,H2Osolub,H2Ores)
ExcessH2O<- H2Ores-H2Osolub

#########CONDITIONS

ResMel2<- subset(ResMel, P>1.9 & P<2.1)
ResMel4<- subset(ResMel, P>3.9 & P<4.1)
ResMel6<- subset(ResMel, P>5.9 & P<6.1)
ResMel8<- subset(ResMel, P>7.9 & P<8.1)

logicH2Ores2<- subset(logicH2Ores, P>1.9 & P<2.1)
logicH2Ores4<- subset(logicH2Ores, P>3.9 & P<4.1)
logicH2Ores6<- subset(logicH2Ores, P>5.9 & P<6.1)
logicH2Ores8<- subset(logicH2Ores, P>7.9 & P<8.1)

ExcessH2O2<- subset(ExcessH2O, P>1.9 & P<2.1)
ExcessH2O4<- subset(ExcessH2O, P>3.9 & P<4.1)
ExcessH2O6<- subset(ExcessH2O, P>5.9 & P<6.1)
ExcessH2O8<- subset(ExcessH2O, P>7.9 & P<8.1)


#######PLOTS

plot(ResMel2, ExcessH2O2, col = "lightcyan2", xlim = c(20, 100), ylim = c(-8, 16), xlab = "Residual Melt (%)",
ylab = "Excess H2O (wt.%)",  cex = 0.2)
points(ResMel4, ExcessH2O4, col = "pale green3", cex=0.2)
points(ResMel6, ExcessH2O6, col = "purple", cex=0.2)
points(ResMel8, ExcessH2O8, col = "pink", cex=0.2)

# Add a legend
legend(73, 16, legend=c("0.19-0.21 GPa", "0.39-0.41 GPa", "0.59-0.61 GPa", "0.79-0.81 GPa"),
      col=c("lightcyan2", "pale green3", "purple", "pink"), pch=19)

plot(ResMel2, logicH2Ores2, col = "lightcyan2", xlim = c(20, 100), ylim = c(2, 20), xlab = "Residual Melt (%)",
ylab = "H2O solubility (wt.%)", cex = 0.2)
points(ResMel4, logicH2Ores4, col = "pale green3", cex=0.2)
points(ResMel6, logicH2Ores6, col = "purple", cex=0.2)
points(ResMel8, logicH2Ores8, col = "pink", cex=0.2)


# Add a legend
legend(73, 19.5, legend=c("0.19-0.21 GPa", "0.39-0.41 GPa", "0.59-0.61 GPa", "0.79-0.81 GPa"),
      col=c("lightcyan2", "pale green3", "purple", "pink"), pch=19)
```
